# In Situ Bonding Regulation of Surface Ligands for Efficient and Stable FAPbI_3_ Quantum Dot Solar Cells

**DOI:** 10.1002/advs.202204476

**Published:** 2022-10-31

**Authors:** Shanshan Ding, Mengmeng Hao, Changkui Fu, Tongen Lin, Ardeshir Baktash, Peng Chen, Dongxu He, Chengxi Zhang, Weijian Chen, Andrew K. Whittaker, Yang Bai, Lianzhou Wang

**Affiliations:** ^1^ Australian Institute for Bioengineering and Nanotechnology The University of Queensland St Lucia Brisbane QLD 4072 Australia; ^2^ School of Chemical Engineering The University of Queensland St Lucia Brisbane QLD 4072 Australia; ^3^ Australian Centre for Advanced Photovoltaics School of Photovoltaics and Renewable Energy Engineering University of New South Wales Sydney NSW 2052 Australia; ^4^ Faculty of Materials Science and Engineering/Institute of Technology for Carbon Neutrality Shenzhen Institute of Advanced Technology Chinese Academy of Sciences Shenzhen 518055 P. R. China; ^5^ Shenzhen Key Laboratory of Energy Materials for Carbon Neutrality Shenzhen 518055 P. R. China

**Keywords:** perovskites, photovoltaic performance, proton exchange control, quantum dots, stability, surface ligands

## Abstract

Quantum dots (QDs) of formamidinium lead triiodide (FAPbI_3_) perovskite hold great potential, outperforming their inorganic counterparts in terms of phase stability and carrier lifetime, for high‐performance solar cells. However, the highly dynamic nature of FAPbI_3_ QDs, which mainly originates from the proton exchange between oleic acid and oleylamine (OAm) surface ligands, is a key hurdle that impedes the fabrication of high‐efficiency solar cells. To tackle such an issue, here, protonated‐OAm in situ to strengthen the ligand binding at the surface of FAPbI_3_ QDs, which can effectively suppress the defect formation during QD synthesis and purification processes is selectively introduced. In addition, by forming a halide‐rich surface environment, the ligand density in a broader range for FAPbI_3_ QDs without compromising their structural integrity, which significantly improves their optoelectronic properties can be modulated. As a result, the power conversion efficiency of FAPbI_3_ QD solar cells (QDSCs) is enhanced from 7.4% to 13.8%, a record for FAPbI_3_ QDSCs. Furthermore, the suppressed proton exchange and reduced surface defects in FAPbI_3_ QDs also enhance the stability of QDSCs, which retain 80% of the initial efficiency upon exposure to ambient air for 3000 hours.

## Graphical abstract

An in situ surface ligand regulation strategy for deliberately controlling protonated‐oleylamine (OAm) dominated surface binding of formamidinium lead triiodide quantum dots (FAPbI_3_ QDs) is demonstrated. The QDs present reduced long‐chain insulating ligand density without compromising their structural integrity, leading to the corresponding QD solar cell a record power conversion efficiency of 13.8% for FAPbI_3_ QDSCs.

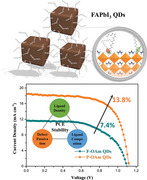

## 1 Introduction

Colloidal perovskite quantum dots (QDs) have demonstrated good potential for photovoltaic applications due to their remarkable tunability in composition and bandgap, small open‐circuit voltage (*V*_oc_) deficit, solution‐processability at low temperatures, as well as chemical and mechanical stability.^[^ ^1^, ^2^, ^3^, ^4^ ^]^ Among the perovskite QD family, formamidinium lead triiodide (FAPbI_3_, FA^+^ = (NH_2_)_2_CH^+^) QDs exhibit superior optoelectronic properties such as desirable bandgap (*E*_g_ = 1.5 eV), prolonged carrier lifetime and excellent phase stability outperforming their inorganic counterparts, e.g. CsPbI_3_ QDs, and hold the promise to further boost the efficiency of quantum dot solar cells (QDSCs).^[^ ^3^, ^5^, ^6^, ^7^, ^8^ ^]^ However, there is a paucity of research on FAPbI_3_ QDSCs, which is in large part due to the difficulty in handling the insulating surface ligands.^[^ ^3^, ^9^ ^]^ Unlike inorganic CsPbI_3_ QDs, the intrinsically weak ionic bonding between the organic cations and inorganic [PbI_6_] octahedral cages in FAPbI_3_ QDs makes them highly susceptible to degradation in polar solvents, which poses great challenges to surface ligand management and defect passivation.^[^ ^3^ ^]^ These negative effects can be partially mitigated by employing additional conjugated small molecules to facilitate the charge extraction in QD devices, however, the reported highest efficiency of FAPbI_3_ QDSCs (power conversion efficiency (PCE) = 12.7%) is still far below their theoretical limit.^[^ ^9^ ^]^ In addition, the use of these small molecules adds cost to the device's fabrication.^[^ ^10^, ^11^ ^]^

The key to resolving this issue in FAPbI_3_ QDSCs lies in the effective management of surface ligands. The surface ligands, generally oleic acid (OA) and oleylamine (OAm), play a critical role in ensuring the colloidal stability and structural integrity of QDs.^[^ ^4^, ^12^ ^]^ Noteworthy that the proton exchange equilibrium between OA and OAm can shift the bonding state of the surface ligands, which is represented by the ratio of oleylammonium (or protonated‐OAm) and free‐OAm on the QD surface.^[^ ^13^, ^14^ ^]^ Protonated‐OAm undergoes strong bonding towards the QD surface, while free‐OAm forms weak bonding to surface iodine or lead ions of QDs and could easily detach from the QD surface upon exposure to polar solvents or moisture in the air.^[^ ^15^, ^16^, ^17^ ^]^ The latter would deteriorate the optoelectronic properties and gradually induce the phase transformation of the QDs during the aging process.^[^ ^14^ ^]^ Moreover, free‐OAm can coordinate with lead species, typically PbX_2_, leading to the formation of (OAm)_2_PbX_4_, which sacrifices the QD stability.^[^ ^18^ ^]^ To date, most efforts in this field have been devoted to exploring alternative ligands to improve the optoelectronic properties and colloidal stability of perovskite QDs, rare attention has been paid to the regulation of the bonding state of their surface ligands.^[^ ^19^, ^20^, ^21^, ^22^, ^23^, ^24^ ^]^ Therefore, an in‐depth understanding of the surface ligand environment (i.e., ligand density and ligand composition) of FAPbI_3_ QDs is still lacking, and strategies to control the bonding state of ligands, as well as their density are highly demanded, which are crucial in tuning their optoelectronic properties and boosting their photovoltaic performance.

In this work, we demonstrate an in situ surface ligand regulation strategy that results in protonated‐OAm dominated surface binding along with reduced long‐chain ligand density for FAPbI_3_ QDs without compromising their structural integrity. It is found that the direct use of protonated‐OAm from oleylammonium iodide during the synthesis of FAPbI_3_ QDs can effectively prevent proton exchange and circumvent the presence of free‐OAm in this system. As a result, the ligand density and defects are significantly reduced in the FAPbI_3_ QDs synthesized in the presence of protonated‐OAm, and their use in solar cells delivers a high PCE of 13.8%, which is a new record for pure FAPbI_3_‐based QDSCs and with a 65% enhancement compared to the previously reported one (without any additive within the light‐harvesting layer).^[^ ^3^ ^]^ Noteworthy that those QD devices also exhibit excellent stability with 80% of the original PCE retained after exposure to ambient air for 3000 hours, thanks to the enhanced surface passivation by suppressing the proton exchange between ligands in the QD solids.

## 2 Results and Discussion

### 2.1 Decoupling Lead and Iodide in the QD Synthesis

To date, most colloidal FAPbI_3_ QDs are prepared via a standard OA/OAm‐based route, in which a dried FA‐oleate solution is swiftly injected into a hot PbI_2_‐1‐octadecene (ODE) solution (with appropriate amounts of OA and OAm) at a specific reaction temperature.^[^ ^3^, ^9^, ^25^ ^]^ However, this OA/OAm‐based system has three major drawbacks. First, as described in Equation 1 and Equation 2, it is difficult to precisely control the reactive molar ratio of lead to iodide due to the fixed ratio in the chosen inorganic lead halide precursors. In this situation, it is problematic to obtain perovskite QDs in a halide‐rich environment, demonstrated to be essential to exclude the formation of halide vacancies and produce perovskite materials with high stability and optical properties.^[^ ^26^, ^27^, ^28^ ^]^ Second, the poor solubility of the metal halide precursors in this ligand/solvent system greatly affects the generalization of this traditional method on the synthesis of colloidal nanocrystals.^[^ ^29^, ^30^ ^]^ A third drawback is related to the overall ligand density. To initiate the nucleation and growth of perovskite QDs, a stoichiometric excess of metal halides relative to FAPbI_3_ QDs is required, which needs excess OA and OAm ligands to dissolve the precursor and confine the particle growth. This can inevitably bring a difficulty to the purification of the products. Consequently, the impaired optoelectronic properties of as‐prepared colloidal QDs would deplete the performance of the corresponding solar cell devices. In this work, control FAPbI_3_ QDs were synthesized via this standard OA/OAm‐based method and named “F‐OAm QDs”, in which F‐OAm means the participation of free‐OAm.

To overcome these drawbacks, in this study, Pb^2+^ and I^−^ sources are independently provided by dissolving lead acetate trihydrate and oleylammonium iodide (OLAI) in OA/ODE and toluene, respectively, to prepare FAPbI_3_ QDs. On the one hand, this method can provide precise control of the I/Pb ratio in the QD synthesis, which should have a positive impact on impeding defect formation.^[^ ^31^ ^]^ On the other hand, the direct use of oleylammonium from OLAI helps suppress proton exchange between OA and OAm, which we believe can form surface ligands with strong binding forces. To verify this, evidence and analysis will be provided in the later sections. The detailed reaction processes are shown in Equations 3 and 4. Upon injection at 80 °C in an N_2_ atmosphere, a rapid salt metathesis reaction occurs, OLAI reacts with the Pb/FA‐oleate to trigger the immediate nucleation and growth of FAPbI_3_ QDs with oleylammonium oleate produced as a by‐product.^[^ ^32^ ^]^ In the following discussion, “P‐OAm QDs” represents samples prepared via this P‐OAm‐based method, in which P‐OAm means protonated‐OAm. In Figure S1 (Supporting Information, SI), we provide a schematic illustration of the synthesis procedure of colloidal FAPbI_3_ QDs.

Synthesis of F‐OAm QDs through a standard OA/OAm‐based route (R means the oleyl groups):

(1) $$\begin{array}{ccc} & & \mathrm{FA}\left({\mathrm{CH}}_{3}{\mathrm{COO}}^{-}\right)+R-\mathrm{COOH}\overset{\Delta }{\to }{\mathrm{FA}}^{+}\left(R-{\mathrm{COO}}^{-}\right)\\ & & \;+\;{\mathrm{CH}}_{3}\mathrm{COOH} \end{array}$$

(2) $$\begin{array}{ccc} & & 2{\mathrm{FA}}^{+}\left(R-{\mathrm{COO}}^{-}\right)+3{\mathrm{PbI}}_{2}+2R-\mathrm{COOH}+R\\ & & \;-\;{\mathrm{NH}}_{2}\overset{\Delta }{\to }2{\mathrm{FAPbI}}_{3}+{\mathrm{Pb}}^{2+}{\left(R-{\mathrm{COO}}^{-}\right)}_{2}\\ & & \;+\;\left(R-{\mathrm{NH}}_{3}^{+}-{}^{-}\mathrm{OOC}-R\right)\mathrm{or}\\ & & {\mathrm{FA}}^{+}\left(R-{\mathrm{COO}}^{-}\right)+{\mathrm{PbI}}_{2}+R-\mathrm{COOH}+R-{\mathrm{NH}}_{2}\overset{\Delta }{\to }{\mathrm{FAPbI}}_{3}\\ & & \;+\;I-\mathrm{vacancy}+\left(R-{\mathrm{NH}}_{3}^{+}-{}^{-}\mathrm{OOC}-R\right) \end{array}$$

Synthesis of P‐OAm QDs through the P‐OAm‐based method:

(3) $$\begin{array}{ccc} & & \mathrm{FA}\left({\mathrm{CH}}_{3}{\mathrm{COO}}^{-}\right)+\mathrm{Pb}{\left({\mathrm{CH}}_{3}{\mathrm{COO}}^{-}\right)}_{2}\cdot 3H_{2}O+3R\\ & & -\;\mathrm{COOH}\overset{\Delta }{\to }{\mathrm{FA}}^{+}\left(R-{\mathrm{COO}}^{-}\right)+{\mathrm{Pb}}^{2+}{\left(R-{\mathrm{COO}}^{-}\right)}_{2}\\ & & +\;3{\mathrm{CH}}_{3}\mathrm{COOH} \end{array}$$

(4) $$\begin{array}{ccc} & & {\mathrm{FA}}^{+}\left(R\;-\;{\mathrm{COO}}^{-}\right)+3R\;-\;{\mathrm{NH}}_{3}^{+}I^{-}+{\mathrm{Pb}}^{2+}{\left(R\;-\;{\mathrm{COO}}^{-}\right)}_{2}\overset{\Delta }{\to }{\mathrm{FAPbI}}_{3}\\ & & \;+\;3\left(R-{\mathrm{NH}}_{3}^{+}-{}^{-}\mathrm{OOC}-R\right) \end{array}$$

### 2.2 Morphology and Optical Properties

**Figure 1:**
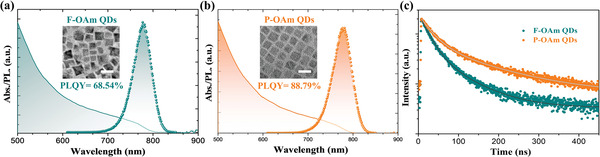
UV‐vis absorption (solid curves), PL spectra (dotted curves), and low‐resolution TEM images (inset, the scale bar is 20 nm) of a) F‐OAm QDs and b) P‐OAm QDs. c) TRPL spectra of as‐prepared FAPbI_3_ QDs and their fitted curves.

To study morphology and optical properties of these FAPbI_3_ QDs, transmission electron microscopy (TEM), photoluminescence (PL), and UV‐vis absorption studies were carried out and the results are shown in **Figure** **1**a,b, and Figure S2, Supporting Information. The reaction temperature of P‐OAm QDs was optimized and more details can be found in the SI. It is found that 80 °C is the optimum reaction temperature for P‐OAm QDs because of the high PL quantum yield (PLQY) and uniform cubic morphology. This is also consistent with the reported FAPbI_3_ QDs synthesized via the standard OA/OAm‐based route.^[^ ^3^, ^33^ ^]^ In addition, compared to the F‐OAm QDs, P‐OAm QDs have more uniform cubic morphology and narrower size distribution of 12.55 ± 1.12 nm. The slightly larger and broader size distribution (14.77 ± 2.16 nm) of F‐OAm QDs may be attributed to the direct participation of OAm in the QD synthesis process, which may extract ions from the QD surface, leading to the regrowth or aggregation of the QDs. The X‐ray diffraction (XRD) patterns in Figure S4a, Supporting Information, show that the as‐synthesized materials are indexed as photo‐active cubic FAPbI_3_.^[^ ^3^ ^]^ No impurity peaks were detected in their XRD patterns, indicating that no crystal structure transformation appeared in these QDs. This is also consistent with the reports that precursor composition variation would not have a significant impact on the phase of perovskite QDs.^[^ ^34^ ^]^ As for the optical properties of these as‐synthesized QDs, as displayed in Figure 1a,b, both F‐OAm QDs and P‐OAm QDs exhibit a PL emission peak and absorption edge at around 790 nm, indicating a near‐infrared absorption and a desirable bandgap (around 1.55 eV, Figure S4b, Supporting Information) for photovoltaic applications. To better understand how proton exchange between ligands effects on the optical properties of QDs, PLQYs of F‐OAm QDs and P‐OAm QDs were analyzed as shown in Figure 1a,b, and Figure S2, Supporting Information. It is found that P‐OAm QDs exhibit a much higher PLQY (88.79%) in contrast to that of F‐OAm QDs (68.54%), which substantiates that the protonated‐OAm can improve the quality and enhance the radiative recombination rate of FAPbI_3_ QD materials. On the other hand, in the standard OA/OAm‐based synthesis system, an excess quality of lead iodide can leave more under‐coordinated lead species on the QD surface and cause a halide‐deficient environment, which eventually sacrifices the optical properties of QDs. While in our P‐OAm‐based QD synthesis system, the fact is that lead acetate trihydrate and oleylammonium iodide were independently used as the lead and iodide sources. Therefore, the improvement of PLQY in P‐OAm QDs should be the combined effects of precise control of the I/Pb ratio and effective surface passivation. This further demonstrates that the precursor composition variation has a positive effect on tuning the optical properties of as‐synthesized perovskite QDs.^[^ ^34^ ^]^ Furthermore, time‐resolved PL (TRPL) decay measurements were employed to elucidate the behavior of charge carriers in these QDs. Their carrier recombination lifetime can be evaluated by fitting the PL decay curves with a bi‐exponential function of time (*t*):

(5) $$F\left(t\right)=A_{1}e^{-\frac{t}{\tau_{1}}}+A_{2}e^{-\frac{t}{\tau_{2}}}+y_{0}$$

where *τ*_1_ and *τ*_2_ are the time constants of the fast and slow decay process, respectively. The fast decay component (*τ*_1_) is attributed to trap‐assisted non‐radiative recombination (interface/surface defects), and the slow component (*τ*_2_) is assigned to radiative recombination.^[^ ^35^ ^]^ According to the results summarized in Figure 1c and Table S1, Supporting Information, P‐OAm QDs exhibit a much prolonged average lifetime of 102.28 ns, which is more than two times as long as that of the F‐OAm QDs (49.76 ns). Such a prolonged lifetime suggests a suppressed trap‐assisted recombination and enhanced quality of P‐OAm QDs.

### 2.3 Control of Surface Ligand Density

**Figure 2:**
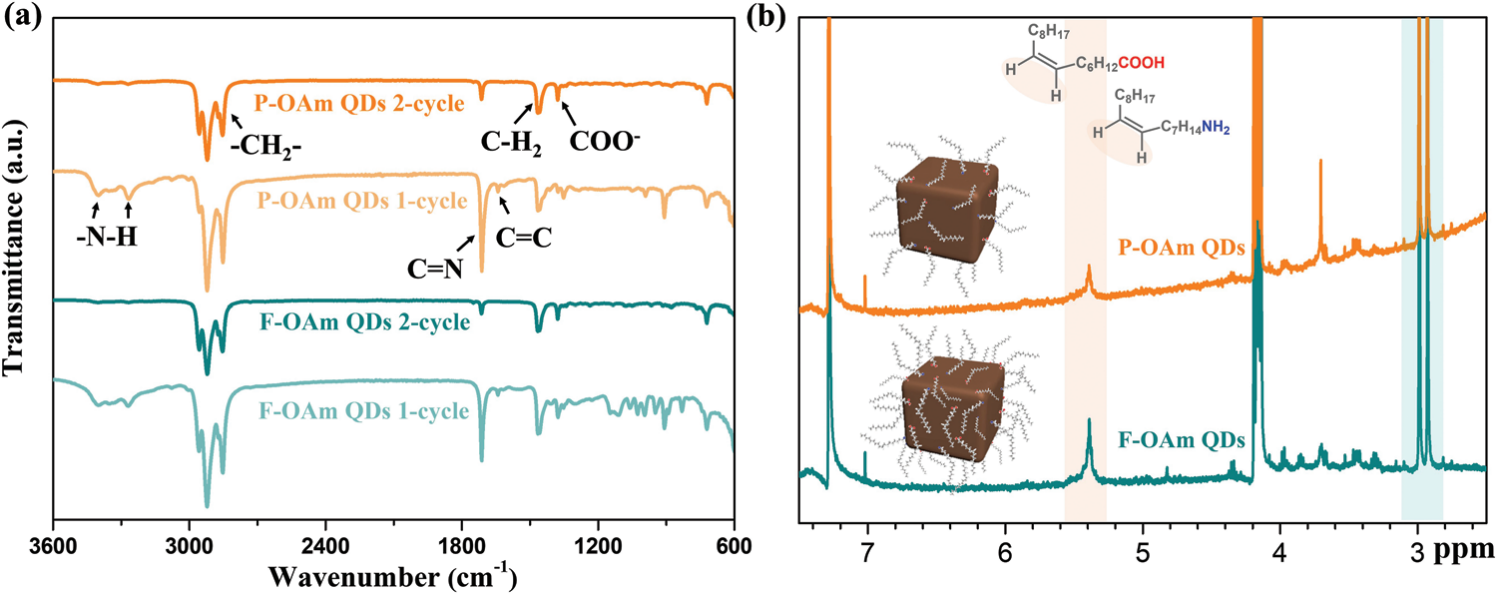
a) FTIR spectra of FAPbI_3_ QDs purified with different cycles. b) ^1^H NMR spectra of as‐prepared FAPbI_3_ QDs after two‐cycle purification. The schematic illustration of different QDs capped with ligands is shown as inset. The functional groups of the oleyl species and their corresponding signals are highlighted with a light orange zone, while the light cyan zone shows the signals of DMF served as a quantitation standard.

The optoelectronic properties of perovskite QDs are closely related to their surface ligand density.^[^ ^4^, ^8^, ^12^ ^]^ Although the long‐chain ligands (e.g., OA and OAm) can serve as defect passivators by coordinating with surface ions of QDs, they impede effective charge extraction and transfer in the resulting QD films due to their insulating nature. Thus, an effective purification process after the QD synthesis is essential to achieve suitable ligand density that guarantees both defect passivation and charge transport efficacy for high‐performing QDSC devices.^[^ ^3^, ^4^ ^]^ Herein, polar solvents, such as methyl acetate (MeOAc), ethyl acetate (EtOAc), and 2‐pentanol, were introduced as anti‐solvents in the purification process of FAPbI_3_ QDs to remove excess long‐chain ligands. It is found that P‐OAm QDs purified with MeOAc, EtOAc or 2‐pentanol have PLQYs of 88.79% (Figure 1b), 72.87% (Figure S5a, Supporting Information), or 53.51% (Figure S5b, Supporting Information), respectively. Such a decrease in PLQY with an increase in polarity of the anti‐solvents is in line with our expectations that high‐polarity solvents could remove more surface ligands and induce more defect formation on the QD surface, thus causing the deterioration of the optical property. It has been reported that 2‐pentanol is effective in improving the charge transport in FAPbI_3_ QDs prepared via the standard OA/OAm‐based method.^[^ ^3^ ^]^ However, in our observation, P‐OAm QDs purified with 2‐pentanol have a lower PLQY than that of F‐OAm QDs (PLQY = 68.54%, Figure 1a) with the same purification condition. This may be due to the lower overall ligand density in P‐OAm QDs compared with that of F‐OAm QDs, making P‐OAm QDs less resistant to the damage caused by such high‐polarity solvent, which will be further discussed in the following part. In this case, to better control the ligand density of P‐OAm QDs without sacrificing their structural stability, MeOAc and EtOAc with a mild polarity were selected as the anti‐solvent for the first and second purification cycles respectively, while 2‐pentanol and EtOAc were used sequentially for the purification of F‐OAm QDs. After each purification process, the density changes of the long‐chain insulating ligands were monitored by the Fourier‐transform infrared (FTIR) spectroscopy. As shown in **Figure** **2**a, the vibration bands at 1640 cm^−1^ and 2500–3000 cm^−1^ are mainly attributed to the oleyl group of OA/OAm ligands. It is found that there is an obvious decrease in intensity with increased purification cycles of FAPbI_3_ QDs, indicating that a large fraction of long‐chain ligands are removed from the QD surface.^[^ ^25^, ^36^ ^]^

To further quantify the surface ligand densities of the as‐synthesized F‐OAm QDs and P‐OAm QDs, ^1^H nuclear magnetic resonance (NMR) measurement was carried out. In Figure 2b (Figure S6, Supporting Information, is the raw data of the corresponding integral area), the strong resonance signal located at around 5.4 ppm (light orange zone) is ascribed to the vinyl protons of the oleyl species (the molecule structure is shown in the inset of Figure 2b) from surface long‐chain OA/OAm ligands. Signals detected at 2.9–3.0 ppm (light cyan zone, Figure 2b) come from the dimethylformamide (DMF), which is served as an internal standard for the quantitation. Interestingly, F‐OAm QDs have a high density of oleyl species on the surface despite that a strong anti‐solvent (2‐pentanol) purification process was employed for the first cycle, as suggested by the relatively stronger NMR signal intensity due to the vinyl protons compared to that of P‐OAm QDs. This also indicates that the low PLQY of F‐OAm QDs described above is not caused by the low surface ligand density but by others. In short, compared to F‐OAm QDs, there is fewer long‐chain insulating ligands on the P‐OAm QD surface, which is the reason why a mild purification process was employed to promise less structural damage and benign electronic coupling property of QDs.

### 2.4 Surface Ligand Composition

**Figure 3:**
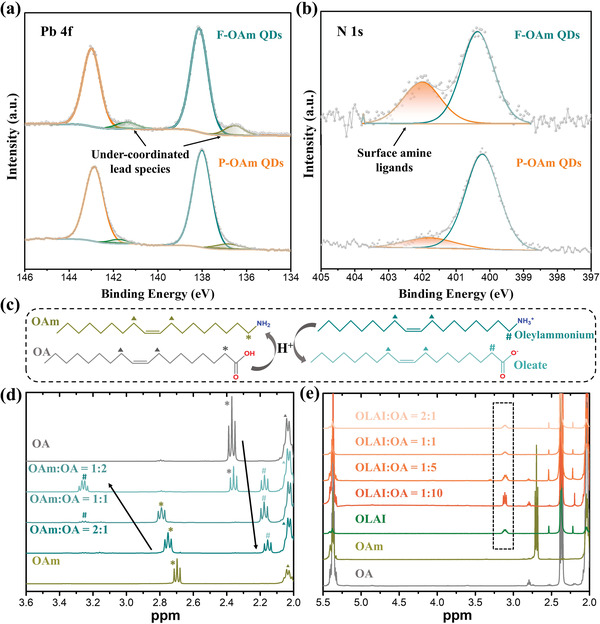
The XPS of a) Pb 4f and b) N 1s core level spectra of different FAPbI_3_ QDs. c) Schematic illustration of the proton exchange between OA and OAm and the molecular structure of OAm, OA, oleylammonium cation, and oleate anion. Selected regions of the ^1^H NMR spectra of d) OAm/OA samples prepared with different molar ratios and e) OLAI/OA samples prepared with different molar ratios. The *α*‐CH_2_ resonances of OAm are signed with merdoie “*” and *α*‐CH_2_ resonances of OA are marked with grey “*”, while “#” means the (de)protonated state of (OA)OAm. Peaks belonging to the *α*‐CH_2_ resonances of oleylammonium cations are highlighted with black dashed rectangular in (e).

It is generally accepted that a high ligand density would be favorable for QDs in the aspect of optical properties.^[^ ^37^ ^]^ However, it is observed that F‐OAm QDs with a higher ligand density exhibit lower PLQY (68.54%) than that of P‐OAm QDs (PLQY = 88.79%). To unveil more details on the improved optical property of P‐OAm QDs, X‐ray photoelectron spectroscopy (XPS) was employed to investigate the surface chemistry of as‐synthesized QDs. The XPS core level spectra of the constituent elements of QDs are shown in **Figure** **3**a,b, and Figure S7, Supporting Information. Compared to the Pb 4f and I 3d spectra of F‐OAm QDs (Figure S7, Supporting Information), the corresponding peaks for P‐OAm QDs slightly shift toward lower binding energy, demonstrating the changed surface ligand environment. Specifically, two extra peaks located at 136.5 eV and 141.4 eV can be observed in the Pb 4f core level spectra (Figure 3a). This is mainly due to the under‐coordinated lead species, which can serve as trap states for the non‐radiative recombination and have a negative impact on the optoelectronic properties of perovskite QDs.^[^ ^26^ ^]^ Noteworthy that F‐OAm QDs have a higher under‐coordinated lead/lead ratio (0.0921) than that of P‐OAm QDs (0.0707), indicating the formation of under‐coordinated lead species is effectively suppressed in P‐OAm‐based system. Considering that the under‐coordinated lead is highly related to the halide vacancy in perovskite QDs, the ratio of I/Pb was also analyzed by XPS and Energy‐dispersive X‐ray spectroscopy (EDS). In the quantitative XPS results (Figures 3a and S7, Supporting Information), F‐OAm QDs have a ratio of I/Pb (2.9542) while P‐OAm QDs have a higher ratio of I/Pb (3.2802) over the stoichiometric value of FAPbI_3_, indicating P‐OAm QDs are with a halide‐rich surface environment. Such a halide‐rich surface can effectively prevent the formation of defects and thus improve the structural stability and optoelectronic performance of perovskite QDs.^[^ ^26^, ^38^ ^]^ This result is further confirmed by the EDS characterization and the resulting patterns are shown in Figure S8, Supporting Information. The formation of a halide‐rich surface is highly related to the applied QD synthesis system here, which will be discussed in the following parts.

Amine ligands, typically OAm and oleylammonium species, have been regarded as the main capping ligands on the QD surface in tuning the dispersibility in solvents and chemical durability of QDs.^[^ ^4^ ^]^ To further analyze the amine species on the QD surface, the N 1s core level spectra were collected. As shown in Figure 3b, the N 1s peak can be fitted into two sub‐peaks located at 400.2 eV and 401.8 eV, ascribing to the FA cations and surface amine complex of QDs.^[^ ^19^, ^39^, ^40^ ^]^ We find that F‐OAm QDs have a higher ratio of surface amine complex/Pb (0.0856) than that of P‐OAm QDs (0.0304), which means that there are more ligands containing amine groups on the F‐OAm QD surface. Generally, in the typical OA/OAm‐based system, oleylammonium oleate and OAm are the main surface capping agents, in which oleate ligands do not directly bind to the QD surface but serve as an ion pair with oleylammonium.^[^ ^17^, ^41^ ^]^ In this regard, the ratios of C/Pb and O/Pb in QD films were also discussed and the results can be found in Figures S9 and S10, Supporting Information. The relatively lower ratio of C/Pb in P‐OAm QDs further confirms that there are fewer long‐chain ligands on the surface, while their slightly higher ratio of O/Pb manifests that P‐OAm QDs are capped with more oleylammonium oleate species on the surface compared to that of F‐OAm QDs. Therefore, given that F‐OAm QDs have a larger amount of oleyl species but a lower density of oleylammonium oleate species than that of P‐OAm QDs based on NMR and XPS results, we can conclude that OAm capping ligands play a dominant role on the F‐OAm QD surface. For the P‐OAm QDs, oleylammonium iodide can directly bind to the QD surface as X‐type ligands, promoting the coordination of the halide anions with positively charged surface centers, ensuring a halide‐rich environment on the QD surface.^[^ ^37^, ^42^ ^]^

To further understand the role of surface ligands on the QD surface, we simulated the dynamic equilibrium between OA and OAm and monitored it with ^1^H NMR technology. Considering the disturbance of FA cations and weak resonance signals of surface ligands, bare OAm/OA and OLAI/OA samples prepared under an N_2_ atmosphere at 80 °C were tested. Meanwhile, because the OA ligands can be effectively removed in the post‐purification processes, a series of OAm/OA or OLAI/OA samples with different molar ratios were prepared and measured by ^1^H NMR technology for a better understanding of the surface ligand composition on QD surface. OAm can be protonated by OA to form oleylammonium and oleate, their molecular structures and corresponding ^1^H NMR response signals are shown in Figure 3c,d. It is observed that the signature of the *α*‐CH_2_ resonances of OAm exhibits a downfield shift with the increased molar ratio of OA in the reaction system, which is the result of the nitrogen protonation. Oppositely, *α*‐CH_2_ resonances of OA show upfield shifts as the carboxylic acid deprotonated. This further verifies that the formation of oleylammonium oleate from the proton exchange between OAm and OA is a dynamic equilibrium (Figure 3c), which is highly related to the initial molar ratio of OAm/OA. Enlighten by these analyses, we can conclude that OAm also plays a significant role on the surface of F‐OAm QDs, which contrasts with the case of P‐OAm QDs. For F‐OAm QDs, the removal of OA and free‐OAm ligands happens easily during the post‐purification process, causing the equilibrium shift toward the reactants (Figure 3d). Therefore, in the post‐ligand treatment or the long‐term storage of F‐OAm QDs, such dynamic equilibrium can accelerate ligand loss and defect formation on the QD surface. Encouragingly, the direct use of protonated‐OAm which replaces OAm molecule can mitigate these negative effects, which is in the case of P‐OAm QDs. As shown in Figure 3e, no obvious peak shift belonging to the *α*‐CH_2_ resonances of oleylammonium is observed no matter how the molar ratio of OLAI/OA increases or decreases, which further demonstrates that the main amine‐based ligands on the P‐OAm QDs are oleylammonium species and the proton exchange between OA and OAm has been suppressed.

To further substantiate the importance of protonated‐OAm in improving the optical properties of P‐OAm QDs, tetra‐n‐octylammonium iodide (TOLI) was used to partially substitute OLAI during the QD synthesis, as TOLI cannot provide protonated amine ligands to QDs due to its large steric hindrance.^[^ ^43^ ^]^ As displayed in the TEM image (Figure S11a, Supporting Information), some irregular shapes and holes can be observed in the structure of resulting FAPbI_3_ QDs. These QDs exhibit a relatively lower PLQY (Figure S11b, Supporting Information) compared to QDs prepared by using OLAI as the pure halide source. Meanwhile, due to the reduced density of oleylammonium species, the structural stability of the resultant OLAI/TOLI‐QDs is rather poor, and thus the purified QDs can be easily aggregated and separated from the non‐polar dispersant (hexane). Therefore, we can conclude that a high ratio of oleylammonium species on the FAPbI_3_ QD surface plays a critical role in tuning the optoelectronic properties and stability of QDs.

### 2.5 Surface Ligand Binding Motifs

**Figure 4:**
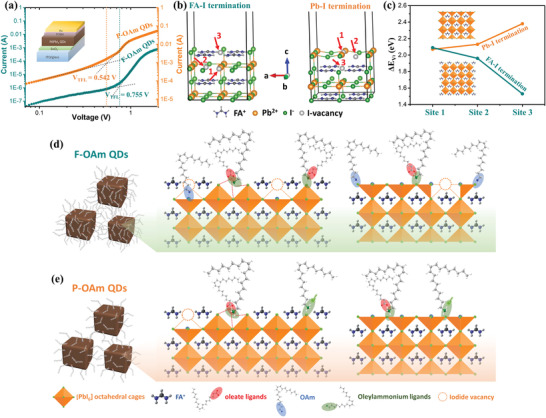
a) *I*–*V* curves of electron‐only devices (ITO/SnO_2_/QDs/PCBM/Au) based on FAPbI_3_ QDs. b) The schematic structure of FA‐I and Pb‐I termination faces for *α*‐FAPbI_3_ materials in the z‐direction (direction of (001) and (002)), the orange and green spheres represent lead and iodine atoms, respectively. To have a clear visualization, four layers are shown in the structure. c) Formation energy of iodide vacancy (*ΔE*_VI_) for *α*‐FAPbI_3_ materials at different potential vacancy sites. Schematic illustration of chemical surface binding motifs for d) F‐OAm QDs and e) P‐OAm QDs dominated with FAI and PbI_2_ termination faces, respectively.

To further confirm the defect reduction of P‐OAm QDs, the space charge‐limited current (SCLC) measurement was applied to study the defect density of QDs. **Figure** **4**a presents the dark *I–V* curves for electron‐only devices with a structure of glass/indium doped tin oxide (ITO)/SnO_2_/FAPbI_3_ QDs/[6,6]‐phenyl‐C61‐butyric acid methyl ester (PCBM)/Au. In all cases, three regions can be observed, which are Ohmic region (a linear relationship between current and electric field) at low bias followed by a nonlinear increase in current as the voltage increases, and a trap‐free region at high bias. Normally, the cross‐over voltage between the first two slopes is the trap‐filled limit voltage (*V*_TFL_), which indicates that the continuously filled trap density levels attain saturation at this stage. According to the *V*_TFL_, the trap density (*N*_t_) of QDs can be further calculated with the following equation:

(6) $$N_{t}=\frac{2\varepsilon \varepsilon_{0}V_{\mathrm{TFL}}}{eL^{2}}$$

where *ε* represents the relative dielectric constant of FAPbI_3_ (*ε* = 46.9),^[^ ^44^ ^]^ *ε*_0_ is the vacuum permittivity (*ε*_0_ = 8.854 × 10^−14^ F/cm), *e* is the elementary charge (*e* = 1.602 × 10^−19^ C) and *L* is the thickness of QD film (*L* = 300 nm). As displayed in Figure 4a, the trap‐filling occurs in relative terms at a higher voltage in F‐OAm QD samples (*V*_TFL_ = 0.755 V) versus P‐OAm QDs (*V*_TFL_ = 0.542 V). Accordingly, the estimated defect density is decreased from 4.349 × 10^16^ to 3.122 × 10^16^ cm^−3^, confirming the lower trap density and better charge transport of the P‐OAm QDs relative to the F‐OAm QDs. We also performed the PL mapping measurements and the captured dynamic fluorescence lifetime images are displayed in Figure S12, Supporting Information. Uniform PL images show that both of F‐OAm QD and P‐OAm QD films are homogeneous with good quality, while the P‐OAm QD film exhibits a longer PL lifetime combined with fewer surface defects than that of F‐OAm QDs, which is also consistent with the TRPL results.

Based on XPS and TRPL results, under‐coordinated lead species (also named I‐vacancy) are the main defects on the FAPbI_3_ QD surface, leading to undesired non‐radiative recombination and poor optical properties. To acquire the fundamental mechanism of the improved optoelectronic properties and suppressed surface defects of P‐OAm QDs, a computational density functional theory (DFT) calculation was conducted. Here, as shown in Figure 4b, the crystal structure of FAPbI_3_ with *α*‐phase was successfully set up, two different cases which may induce the formation of under‐coordinated lead species in terms of surface binding motifs for F‐OAm QDs/P‐OAm QDs were taken into consideration. One is the FA‐I termination surface and the other is the Pb‐I termination surface. The considered surfaces are presented on the (001) surface of perovskite.^[^ ^45^ ^]^ We first calculate the formation energy of different terminated surfaces and find that the Pb‐I termination surface is slightly more stable (0.031 eV per unit cell) than that of the FA‐I termination surface. Moreover, we calculated the formation energy of iodide vacancy (*ΔE*_VI_) in the structure of FA‐I and Pb‐I termination faces for *α*‐FAPbI_3_ material at different potential vacancy sites (Figure 4b). The vacancy formation energy of these sites is listed in Figure 4c and Table S2 (Supporting Information), in which higher formation energy means that it is less likely to form iodide vacancy at that site. The formation energy of site 2 and site 3 in Pb‐I termination is higher than that of candidate sites in FA‐I termination, suggesting that less iodide vacancy can be formed at Pb‐I termination faces.

Combining all the analysis results, we can deduce the chemical surface binding motifs of FAPbI_3_ QDs synthesized in the two different systems. The most important difference between these two systems is the binding species in the ligand shell (e.g., OAm and oleylammonium oleate species). In the typical OA/OAm‐based system, as displayed in Figure 4d, excess PbI_2_ is used to serve as lead and halide sources simultaneously, which not only leads to the hardly controlled reactive molar ratio of lead to iodide in the final QD structure but also easily induces an iodide deficit environment on the QD surface, resulting in more under‐coordinated lead atoms served as the charge recombination center on the QD surface. Thus, in the case of FA‐I terminated QD surface, although these under‐coordinated lead atoms on the QD surface can be temporarily passivated by OAm ligands to maintain good optical properties, OAm ligands are easily removed during the post‐purification process due to their weak bonding to QD surface. On the other hand, considering the proton exchange between OA and OAm can form oleylammonium oleate species as capping ligands, the removal of OAm ligands can also promote the chemical equilibrium to shift from bounded ligands to free ones, leaving more iodide vacancies on the QD surface and thus causing deterioration in optical property. On the country, in our P‐OAm‐based system (Figure 4e), the employment of OLAI can precisely control the I/Pb ratio in the resulting QDs and impedes the formation of surface under‐coordinated lead atoms. The oleylammonium species can coordinate with surface iodide or partially substitute FA cations. The H atoms in the –NH_3_^+^ moiety can bind with the surrounding iodide on the QD surface by forming H∙∙∙I hydrogen bonds, while oleate or excess iodide species can neutralize the surface charge of QDs, preserving a halide‐rich environment on the P‐OAm QD surface. Similarly, in the case of Pb‐I termination face, although fewer I‐vacancies can be formed due to their relatively higher formation energy, the weak bonding between OAm ligands and surface iodine of F‐OAm QDs would induce the defect formation (site 1, Figure 4b,c) during the post‐purification or ligand‐exchange treatments. This negative effect can be mitigated in the P‐OAm‐based system, in which oleylammonium species are the main surface capping ligands and can promote the coordination of the halide anions with positively charged surface centers, ensuring a halide‐rich environment and reduced defects on the P‐OAm QD surface. Meanwhile, the coordination of oleylammonium species with the P‐OAm QD surface circumvents the proton exchange between OA and OAm, providing QDs with a relatively stable binding surface. Benefiting from the suppressed proton exchange and firm surface binding motif of oleylammonium species, P‐OAm QDs exhibit excellent optoelectronic properties accompanied by reduced non‐radiative recombination.

### 2.6 QD Device Performance

**Figure 5:**
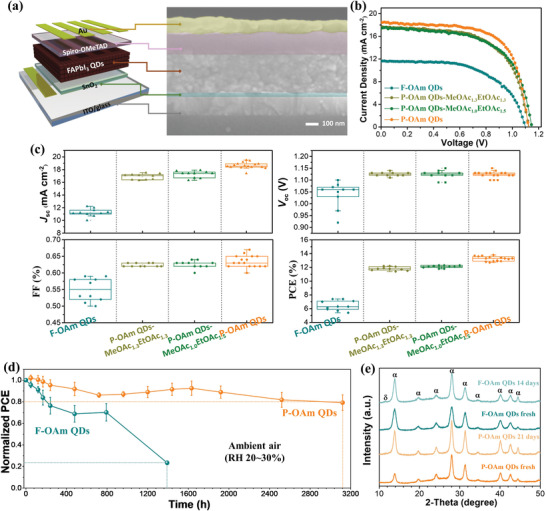
a) Device structure and cross‐sectional SEM image of the as‐fabricated FAPbI_3_ QDSCs. b) Typical *J–V* curves of the champion QDSCs based on as‐prepared FAPbI_3_ QDs. c) Performance evolution of solar cells based on as‐prepared FAPbI_3_ QDs (ten devices for F‐OAm QDs, P‐OAm QDs‐MeOAc_1.3_EtOAc_1.3,_ and P‐OAm QDs‐MeOAc_1.0_EtOAc_1.5_, while sixteen devices for target P‐OAm QDs). The solid dots represent the original data. d) Long‐term stability of unencapsulated QDSCs based on F‐OAm QDs and P‐OAm QDs. Each average (symbol) and standard deviation (error bar) were analyzed based on three device‐testing results. All the devices were stored in ambient air with a RH of 20%–30%. e) The XRD patterns of F‐OAm QDs and P‐OAm QDs measured before and after storage. *α* means the perovskite phase and *δ* manifests the hexagonal non‐perovskite phase.

To verify the appealing photovoltaic performance of P‐OAm QDs, we fabricated solar cells with a device structure of glass/ITO/SnO_2_/FAPbI_3_ QDs/2,2′,7,7′‐tetrakis(*N*,*N*‐*di*‐*p*‐methoxyphenyl amino)‐9,9′‐spirobifluorene (Spiro‐OMeTAD)/Au via a layer‐by‐layer deposition strategy in ambient air (relative humidity level (RH) is 30%–40%, Figure S1, SI). SnO_2_ and Spiro‐OMeTAD are used as electron and hole transport materials, respectively. Each constituent layer of the device can be resolved in the cross‐sectional scanning electron microscopic (SEM) image (**Figure** **5**a). The thickness of the active QD layer is around 300 nm. To mitigate the insulating barrier caused by long‐chain ligands, one more ligand‐exchange steps during the QD film deposition were carried out. MeOAc was used to rinse the QD film and remove the original long‐chain ligands, while the saturated formamidinium iodide (FAI)‐EtOAc treatment was applied to improve the inter‐dot electronic coupling of QD films.^[^ ^20^ ^]^ However, the same ligand‐exchange treatment is not applicable in the case of F‐OAm QD films, because the underlying QD film can be further dissolved by the solvent of the subsequent layer, leading to a non‐uniform QD film with poor quality (Figure S13, Supporting Information). Therefore, EtOAc with a slightly higher polarity was applied to soak the F‐OAm QD film to achieve a high‐performing solar cell device, a strategy also has been demonstrated by Xue et al.^[^ ^3^ ^]^ The detailed device fabrication process is presented in the Experimental Section (SI).

The performance and stability of QDSCs are highly related to the surface insulating barrier and defects of perovskite QDs, while the post‐purification process can inevitably cause defect formation. In this case, the device optimization was performed by tuning the volume ratio of the anti‐solvents (MeOAc or EtOAc)/P‐OAm QDs during the purification process. Based on our previous work, this ratio of anti‐solvents/QDs should be at least >1 for efficient charge transfer.^[^ ^8^ ^]^ Here, the “P‐OAm QDs‐MeOAc_1.3_EtOAc_1.3_” means the resulting P‐OAm QDs were purified by MeOAc in the first cycle with a ratio of 1.3 (MeOAc/QDs, v/v = 1.3) followed by the EtOAc treatment with a ratio of 1.3 (EtOAc/QDs, v/v = 1.3) in the second cycle. Similarly, the “P‐OAm QDs‐MeOAc_1.0_EtOAc_1.5_” illustrates the as‐synthesized P‐OAm QDs were purified with MeOAc (MeOAc/QDs, v/v = 1.0) firstly and EtOAc with a higher ratio (EtOAc/QDs, v/v = 1.5) was carried out in the second cycle. The *J–V* characteristic curves of the best‐performing QDSCs with different purification conditions are emphatically displayed in Figure 5b and the corresponding PV parameters are listed in **Table** **1**. Figure 5c presents the detailed performance statistics of as‐fabricated FAPbI_3_ QDs‐based devices, while the corresponding current density‐voltage (*J–V)* curves of these devices are shown in Figure S14, Supporting Information. All the devices were measured in reverse scan under AM1.5G, 100 mW cm^−2^ simulated solar illuminations. As a control sample, solar cells based on the F‐OAm QDs were also fabricated to investigate the effect of surface ligand regulation on the photovoltaic performance of QDSCs. It is found that the control device exhibits a low short‐circuit current (*J*_sc_) of 11.6 mA cm^−2^, open‐circuit voltage (*V*_oc_) of 1.10 V, and fill factor (*FF*) of 0.58, yielding a PCE of 7.4%. As for the target devices based on P‐OAm QDs, optimal results were obtained using MeOAc with a ratio of 1 in the first cycle followed by EtOAc (EtOAc/QDs, v/v = 1.3) washing process. The champion device delivered a promising PCE of 13.8% (a stabilized PCE of 13.76%, Figure S15a, Supporting Information) with a high *J*_sc_ of 18.5 mA cm^−2^, *V*_oc_ of 1.12 V, and *FF* of 0.67, which is a new record efficiency for pure FAPbI_3_‐based QDSCs.^[^ ^3^, ^9^, ^19^ ^]^ The *J–V* curves measured at the reverse and forward scanning directions were also collected and shown in Figure S15b. Moreover, as illustrated in Figure 5c and S14d, 16 individual P‐OAm QDs‐based cells fabricated from different batches were used for statistics. These P‐OAm QDs‐based solar cells delivered an average PCE of 13.26 ± 0.327%, *J*_sc_ of 18.63 ± 0.507 mA cm^−2^, *V*_oc_ of 1.12 ± 0.014 V, and *FF* of 0.63 ± 0.018, demonstrating a good reproducibility. Such improvements in performance and reproducibility are the result of the decreased surface defects and enhanced charge carrier transfer property. In this regard, we evaluated the ligand density of F‐OAm QD and P‐OAm QD films via FTIR and ^1^H NMR measurements as shown Figures S16 and S17, Supporting Information. It is found that there is a weak FTIR resonance from oleyl species detected in the QD film samples, which manifests that long‐chain ligands have been dramatically removed from the QD surface after ligand‐exchange treatment during the QD film deposition. Moreover, P‐OAm QD films have less long‐chain ligand density than F‐OAm QD films on basis of ^1^H NMR results, yielding a higher *J*_sc_ of P‐OAm QD‐based device. Compared to the optimal FAPbI_3_ QDSCs, the PCE of devices based on “P‐OAm QDs‐MeOAc_1.3_EtOAc_1.3_” and “P‐OAm QDs‐MeOAc_1.0_EtOAc_1.5_” light absorbers had been dramatically decreased, especially *J*_sc_ and *FF*. This should be the result of insufficient removal of long‐chain insulating surface ligands and increased non‐radiative recombination induced by the immoderate removal of ligands, respectively.

**Table 1:** PV parameters extracted from the *J–V* curves of best‐performing QDSCs based on as‐prepared FAPbI_3_ QDs

Samples		*J_sc_* [mA cm^−2^]	*V_oc_* [V]	*FF*	*PCE* [%]
F‐OAm QDs	Champion	11.6	1.10	0.58	7.4
	Average	11.22 ± 0.629	1.04 ± 0.055	0.55 ± 0.035	6.46 ± 0.720
P‐OAm QDs ‐MeOAc_1.3_EtOAc_1.3_	Champion	17.5	1.13	0.62	12.2
	Average	16.89 ± 0.448	1.12 ± 0.008	0.62 ± 0.005	11.82 ± 0.282
P‐OAm QDs‐MeOAc_1.0_EtOAc_1.5_	Champion	17.4	1.15	0.62	12.3
	Average	17.25 ± 0.534	1.12 ± 0.019	0.62 ± 0.012	12.1 ± 0.189
P‐OAm QDs	Champion	18.5	1.12	0.67	13.8
	Average	18.63 ± 0.507	1.12 ± 0.014	0.63 ± 0.018	13.26 ± 0.327

All devices were measured in reverse scan with AM1.5G, 100 mW cm^−2^ simulated solar illuminations.

The SCLC results demonstrate that the decreased *FF*s are attributed to the increase of defect density for the “P‐OAm QDs‐MeOAc_1.3_EtOAc_1.0_” and “P‐OAm QDs‐MeOAc_1.0_EtOAc_1.5_” samples (Figure S18, SI). These results were also confirmed by TRPL characterization. As shown in Figure S19 and Table S1, Supporting Information, the average carrier lifetimes (*τ*_ave_) of “P‐OAm QDs‐MeOAc_1.3_EtOAc_1.3_” and “P‐OAm QDs‐MeOAc_1.0_EtOAc_1.5_” are 65.96 ns and 84.99 ns. Both are slightly lower than that of optimal P‐OAm QDs (*τ*_ave_ = 102.28 ns), indicating that there is more non‐radiative recombination in the QD film and thus causing the carrier quenching. Combining SCLC and TRPL results, we can conclude that the optimal purification ratio of QD/anti‐solvents plays another significant role to achieve good performance in FAPbI_3_ QDSCs apart from ligand binding regulations. A high ratio of polar solvents can easily cause defect formation during the purification process. To further investigate the charge transport dynamics at the interface of QD and ETL layers, we deposited different QD films on top of SnO_2_ layers and collected their TRPL spectra. As illustrated in Figure S20, Supporting Information, all samples exhibit a dramatically decreased PL lifetime when QD films are contacted with the ETL layer, confirming the efficient injection of electrons from the FAPbI_3_ QD layer to the ETL layer. The inset table lists the corresponding parameters of TRPL spectra. The PL lifetime of the F‐OAm QDs/ETL sample is 11.66 ns, while a shorter lifetime of 8.88 ns is obtained in the P‐OAm QDs/ETL sample. This indicates that a faster electron extraction is achieved at the interface of P‐OAm QDs/SnO_2_ due to the reduced interfacial non‐radiative recombination.

As a widely known fact, FAPbI_3_ bulk material has poor phase stability since its desired black *α*‐phase can be easily transformed into yellow *δ*‐phase (non‐perovskite) even at room temperature. Although the tensile surface strain on perovskite QDs can mitigate such phase transformation, the instinct ionic nature and highly dynamic binding character of perovskite QDs still can cause their instability, especially under the attack of ambient stimuli (e.g., water and oxygen). To explore the stability of our QDSCs, we monitored the efficiency change of unencapsulated devices stored in dark ambient air (RH = 20%, 20 °C). As shown in Figure 5d, P‐OAm QDs‐based devices exhibit excellent stability with 80% of the original PCE retained after exposure to ambient air for 3000 hours, while only around 20% of the initial PCEs was retained for the F‐OAm QDs‐based devices after 1400 h. The slight increase of PCE for P‐OAm QDs‐based devices during the aging time is possibly due to the intrinsic ion migration, during which the bulk defects gradually transfer to the QD surface and can further be electronically passivated by surface ligands. The device stability under continuous one‐sun illumination was also evaluated and the normalized PCE change of the unencapsulated devices can be found in Figure S21, Supporting Information. Ex‐situ XRD results (Figure 5e) also provide solid evidence for the improved phase stability of P‐OAm QDs. After the XRD test as fresh samples, these QD films were kept in ambient conditions with around 45% RH at room temperature. After exposure to air for 14 days, there is a peak belonging to the hexagonal non‐perovskite *δ*‐phase observed in the XRD pattern of the F‐OAm QDs, whereas no secondary phase formed in P‐OAm QD sample even after storage for 21 days. The enhanced phase stability of P‐OAm QDs is mainly due to the reduced surface defects and the suppressed proton transfer between OA and OAm, which lessen the transport channel for water molecules and prevent the phase degradation of perovskite cores.

## 3 Conclusion

In summary, we demonstrated an in situ surface ligand regulation strategy by directly introducing protonated‐OAm from OLAI during the synthesis of FAPbI_3_ QDs. The resulting P‐OAm QDs with a controllable size exhibited excellent optoelectronic properties. Combined with FTIR, ^1^H NMR, and XPS results, P‐OAm QDs have a lower long‐chain ligand density than that of F‐OAm QDs, which is beneficial to enhance the electrical coupling in the resulting QD solids. In addition, oleylammonium species play a key role on the P‐OAm QD surface, while OAm is the main amine species bound to the F‐OAm QD surface. Owing to the benefits of the strong binding motif and suppressed proton exchange between OA and OAm, the formation of iodide vacancies on the P‐OAm QD surface can be effectively impeded, resulting in its longer PL lifetime. As a result, the P‐OAm QDs‐based solar cells deliver a high PCE of 13.8%, a record efficiency for pure FAPbI_3_‐based QDSCs to date. Encouragingly, the QD devices also exhibit excellent stability with 80% of the original PCE retained after exposure to ambient air for 3000 hours because of the suppressed proton exchange and reduced non‐radiative recombination in QD solids. We believe this work reveals the significance of in situ ligand bonding regulation in tuning the optoelectronic properties of QDs and has paved a fundamental way to improve the efficiency and enhance the stability of perovskite QDSCs. With the continuing development of advanced ligand management strategies, especially in the construction of functional and conductive ligand shells, the potential for high‐efficient and low‐cost FAPbI_3_ QDSCs will be witnessed.

## Conflict of Interest

The authors declare no conflict of interest.

## Supporting information

Supporting Information

## Data Availability

The data that support the findings of this study are available from the corresponding author upon reasonable request.
