# Supplementary material for: In Situ Bonding Regulation of Surface Ligands for Efficient and Stable FAPbI3 Quantum Dot Solar Cells
Source: Adv Sci (Weinh). 2022 Oct 31;9(35):2204476. doi: 10.1002/advs.202204476 (PMC9762318; doi:10.1002/advs.202204476)
Supplement: Supplementary file 1 — Supporting Information [file ADVS-9-2204476-s001.pdf]

ADVANCED SCIENCE
Open Access

Supporting Information

for *Adv. Sci.*, DOI 10.1002/advs.202204476

In Situ Bonding Regulation of Surface Ligands for Efficient and Stable $FAPbI_3$ Quantum Dot Solar Cells

Shanshan Ding, Mengmeng Hao, Changkui Fu, Tongen Lin, Ardeshir Baktash, Peng Chen, Dongxu He, Chengxi Zhang, Weijian Chen, Andrew K. Whittaker, Yang Bai* and Lianzhou Wang**

In-situ Bonding Regulation of Surface Ligands for Efficient and Stable $FAPbI_3$ Quantum Dot Solar Cells

Shanshan Ding[a], Mengmeng Hao,[a] Changkui Fu[a], Tongen Lin[a], Ardeshir Baktash[a], Peng Chen[a], Dongxu He[a], Chengxi Zhang[a], Weijian Chen[c], Andrew K. Whittaker[a], Yang Bai,[b]* Lianzhou Wang[a]**

[a] Australian Institute for Bioengineering and Nanotechnology and School of Chemical Engineering, The University of Queensland, St Lucia, Brisbane, QLD 4072, Australia

[b] Faculty of Materials Science and Engineering/Institute of Technology for Carbon Neutrality, Shenzhen Institute of Advanced Technology, Chinese Academy of Sciences, Shenzhen 518055, China; Shenzhen Key Laboratory of Energy Materials for Carbon Neutrality, Shenzhen 518055, China.

[c] Australian Centre for Advanced Photovoltaics, School of Photovoltaics and Renewable Energy Engineering, University of New South Wales, Sydney, NSW 2052, Australia

*Corresponding authors and E-mail:

Lianzhou Wang: l.wang@uq.edu.au; Yang Bai: y.bai@siat.ac.cn;

Mengmeng Hao m.hao1@uq.edu.au

Experimental details

Chemicals

Oleic acid (OA, technical grade 90%), oleylamine (OAm, technical grade 70%), 1-octadecene (ODE, technical grade 90%), lead (II) acetate trihydrate ($Pb(Ac)_2 \cdot 3H_2O$, 99.999%), formamidine acetic acid salt (FAAc, ≥99%), toluene (anhydrous, 99.8%), acetonitrile (anhydrous, 99.8%), hexane (reagent grade ≥95%), octane (anhydrous, ≥99%), methyl acetate (MeOAc, anhydrous, 99.5%), ethyl acetate (EtOAc, anhydrous, 99.5%), 2-pentanol (98%), (lead (II) nitrate ($Pb(NO_3)_2$, 99.999%), formamidinium iodide (FAI, anhydrous, ≥99%), chlorobenzene (anhydrous, 99.8%), 4-tert-butylpyridine (TBP, 96%), chloroform-d (99.8 atom % D), N,N-Dimethylformamide, N,N-Dimethylformamide-d_7 (≥99.5) and bis(trifluoromethane) sulfonimide lithium salt (Li-TFSI). All chemicals were purchased from Sigma Aldrich and directly used. Lead (II) iodide (PbI_2 99.9985%) and SnO_2 nanoparticle dispersion (15% in H_2O,) were got from Alfa Aesar. Oleylamonium iodide (OLAI, ≥99%, Xi'an BaOAmIte) and (2,2',7,7'- Tetrakis(N,N-di-p-methoxyphenylamine)-9,9-spirobifluorene (Spiro-OMeTAD, ≥99.8%, Xi'an BaOAmIte) were used as received without further purification.

Synthesis of $FAPbI_3$ QDs

F-OAm QDs (Standard OA/OAm-based route)

The method used in this work for the synthesis of F-OAm QDs was based on Yang‘s previous work with little modification.[1]

Preparation of FA-oleate precursors: 0.521 g (5 mmol) of FAAc were added to a 50 mL three-neck round-bottom flask containing 10 mL OA and degassed under vacuum for 1 h at 40 °C. After that, gradually raise the temperature to 130 °C to obtain a clear solution of FA-oleate under argon atmosphere. The resulting solution was then maintained at 60 °C under Ar for the following reaction.

Synthesis of F-OAm QDs: 0.43 g (0.935 mmol) of PbI_2 were added to a 50 mL three-neck round-bottom flask containing 25 mL of ODE, 5 mL of OA and 2.5 mL OAm, and degassed under vacuum for 1 h at 120 °C until all PbI_2 was dissolved to obtain a clear solution. After that, gradually decrease the temperature to 80 °C under argon atmosphere. And then, 5 mL of FA-oleate was swiftly injected and reacted for around 20 s, followed by an ice bath to cool the mixture.

Purification of F-OAm QDs: 36 mL of 2-pentanol was added in the crude $FAPbI_3$ QDs solution followed by centrifugation at 8000 rpm for 5 min. After that, the precipitate was

washed again with EtOAc (QD:EtOAc = 1:1.3) by centrifugation at 7800 rpm for 4 min. Keep the precipitate dispersed in octane. Before QD film deposition, the QD solution was centrifuged at 3000 rpm for 2 min to prevent the interference of excess salts and aggregated $FAPbI_3$ nanoparticles. The whole process has been depicted in Figure S1.

P-OAm QDs (P-OAm-based method)

Preparation of FA/Pb-oleate precursors: 0.157 g (1.5 mmol) of FAAc and 0.152 g of $Pb(Ac)_2 \cdot 3H_2O$ (0.4 mmol) were added to a 50 mL three-neck round-bottom flask containing 16 mL of ODE and 4 mL of OA, and degassed under vacuum for 1 h at 40 ℃.

Synthesis of P-OAm QDs: gradually raising the temperature of FA/Pb-oleate precursors to 80 ℃ to obtain a clear solution under argon atmosphere. After that, 4 mL of OLAI-toluene (0.474 g of OLAI was dissolved in 4 mL of toluene) solution was swiftly injected and reacted for around 20 s, an ice bath was needed to cool the mixture.

Purification of P-OAm QDs: 24 mL of MeOAc was added in the crude P-OAm QDs solution (QD:MeOAc = 1:1) followed by centrifugation at 7800 rpm for 4 min. The precipitate was redispersed in 5 mL of hexane and washed again with EtOAc (QD:EtOAc = 1:1.3). After centrifuging for 4 min at 7800 rpm, the precipitate was dispersed in octane for QD film deposition. To prevent the interference of excess salts and aggregated $FAPbI_3$ nanoparticles, a further centrifugation step at 3000 rpm for 2 min for the QD solution was needed. The whole process has been depicted in Figure S1.

$FAPbI_3$ QD film fabrication:

For ligand-exchange during film deposition process, saturated solutions of $Pb(NO_3)_2$-MeOAc and FAI-EtOAc were prepared via dissolved 15 mg of salts into 15 mL of polar solvents followed by sonication for 20 min. Centrifugation at 3000 rpm for 3 min was carried out to remove excess salts.

F-OAm QD films: F-OAm QDs ink with a concentration of 80 mg/mL was spin-cast on the SnO_2 substrate at 3000 rpm for 30 s, and then soaked twice in the neat EtOAc solution. These procedures were repeated four times to build appropriate thickness. Finally, the films were dipped into the FAI-EtOAc solution for 5 s followed by soaking in the neat MeOAc and drying.

P-OAm QD films: P-OAm QDs ink with a concentration of 80 mg/mL was spin-cast on the SnO_2 substrate at 3000 rpm for 30 s, and then swiftly soaked in the $Pb(NO_3)_2$-MeOAc solution. In order to remove excess lead salts attached to the surface of QDs, the neat MeOAc was used to rinse the as-treated QDs films. These procedures were repeated four times to

build appropriate thickness. Finally, the films were dipped into the FAI-EtOAc solution for 5 s followed by soaking in the neat MeOAc and drying.

Device fabrication

The whole process was done in ambient air (the relative humidity was 30-40 %). Glass/indium doped tin oxide (ITO) substrates were washed with successive sonication in ethanol, acetone and 2-propanol each for 20 min, respectively. After that, the cleaned substrates were treated in an ultraviolet-ozone chamber before use. The SnO_2 colloidal solution was diluted by deionized (DI) water to 2.6 % and spin-coated onto glass/ITO substrates at 3000 rpm for 30 s followed by baking on a hot plate at 150 °C for 30 min to form dense electron transport layers. As for the photoactive QDs layers, please see the film fabrication. For the hole transfer material, the spiro-MeOTAD solution was prepared by dissolving 75.3 mg of spiro-MeOTAD in 1 mL of chlorobenzene followed by adding 28.8 µL of TBP and 17.5 µL of Li-TFSI (520 mg/mL in acetonitrile) solution. The hole transfer layer was obtained via spin-coating the as-prepared spiro-MeOTAD solution onto the QDs layers at 4000 rpm for 30 s. Finally, the gold electrode was thermally evaporated at 0.5 Å/s with a total thickness of 80 nm to complete the devices. The whole process has been vividly depicted in Figure S1.

Characterizations

Material characterization

The powder X-ray diffraction (XRD) with Cu Kα radiation was used to identify the composition and crystalline phase of prepared samples. Fourier transform infrared spectra (FTIR, Nicolet 5700) were collected to demonstrate the functional groups of PQDs solution. Field Emission Scanning Electron Microscope (SEM, JEOL JSM-7100F), transmission electron microscope (TEM, Hitachi HT 7700) were used to identify the morphology and crystal structure. The Energy-dispersive X-ray spectra (EDS) for elemental analysis were carrier on the Hitachi SU3500-A SEM fitted with an Oxford Xmax SDD EDS detector. The chemical composition was investigated by Kratos Axis Ultra XPS with Al Kα radiation source. Ultraviolet-visible adsorption spectra (Jasco V670) for colloidal solutions was performed to analyse the photo-physical properties. The steady-state PL emissions of QD solutions was measured at 450 nm light source excitation using a monochromatized Xe lamp, and the time-resolved PL (TRPL) decay studies were carried out with a 377 nm pulsed diode laser excitation source on a fluorescence spectrophotometer (FLSP-900, Edinburgh Instruments). As for the PLQY measurements, the as-synthesized QDs were dissolved in

hexane and were estimated by comparing the QD emission with that of an organic dye (methylene blue in ethanol, QY of 52 %) according to standard procedure.[2-4] The PL mapping of QD films were conducted on a confocal laser scanning microscopy equipped with a 479 nm pulsed laser and TCSPC module (MT200, Picoquant). The ^{1}H NMR spectra were collected in solution of chloroform-d on a Bruker AVANCE 400 MHz spectrometer. The NMR samples were prepared as follows. First, 0.2 μL N,N-dimethylformamide (DMF, Sigma-Aldrich, anhydrous, 99.8 %) was added in 1.2 mL chloroform-d. Then, 75 μL of the as-prepared $FAPbI_3$ QD inks diluted with octane to 8 mg/mL was dispersed into the above solutions followed by thoroughly shaking. As for the QD film samples after ligand-exchange treatment, glass/QD films (four layers) was disolved in 0.6 mL of N,N-Dimethylformamide-d_7. To quantify the surface ligand density, 8 μL of ethyl acetate/N,N-Dimethylformamide-d_7 (v/v = 1/1000) was added as a standard.

Device characterization

The current density-voltage (*J-V*) curves of the as-fabricated devices were recorded with a source meter (Keithley 2420) using a solar simulator (Newport, Oriel Class AAA, 94063A) at 100 mW/cm^2 illumination (AM 1.5 G) equipped with a calibrated silicon reference cell and meter (Newport, 91150V) certificated by the National Renewable Energy Laboratory (NREL). The *J-V* curves were measured in reverse scan (from 1.3 V to -0.1 V) modes at a scan speed of 100 mV/s with a delay time of 100 ms. A metal shadow mask with the area of 0.08 cm^2 was employed to define the active area of devices. All as-fabricated devices measured directly without any encapsulation in ambient air. The space charge-limited current (SCLC) measurements were conducted on an electrochemical workstation (CHI 660E, Shanghai Chenhua Instruments, China).

Computational Methodology

$FAPbI_3$ with ideal α-phase was energy optimised, two surfaces were considered as our initial possible surfaces. Both structures were geometry optimised and their energies compared. To study the formation of iodine vacancy in the structure of α-$FAPbI_3$ perovskite, density functional theory (DFT) as implemented in the Vienna Ab Initio Package (VASP) is applied.[5] For all the calculations, the generalized gradient approximation (GGA) with the use of Perdew− Burke−Ernzehof (PBE) functional is applied to adopt the exchange-correlation effect.[6] To correct the Van der Waals interactions a D3-BJ dispersion correction is applied.[7,

8] To describe the core and valence electrons, the projector augmented wave (PAW) method with an energy cutoff of 520 eV and a force tolerance of 10^{-4} eV $Å^{-1}$ is applied.

For the bulk structure a 3 × 2 × 2 supercell (144 atoms) is modelled and to model the surface of α-$FAPbI_3$ perovskite structure, a 3 × 2 six-layers slab (144 atoms) is considered. The following equation is used to calculate the formation energy of the iodide vacancy in the structure of α-$FAPbI_3$ perovskite.

$$\Delta E_{\mathrm{VI}} = E_{\mathrm{Str\text{-}v}} + \mu_{\mathrm{I}} - E_{\mathrm{pure}} \qquad \text{(S1)}$$

where ΔE_{VI} is the formation energy of iodide vacancy, $E_{\mathrm{Str\text{-}v}}$ is the total energy of structure with iodide vacancy, μ_{I} is the chemical potential of iodine, E_{pure} is the total energy of the un-doped structure. To model the surface, a constraint has been applied to the surfaces. To converge the surfaces, the atoms at the top layer (top four layers) are left to optimise and atoms from the inner layers (two bottom layers) are considered fixed. To model surface, a vacuum of 20 Å is considered in *z* direction.

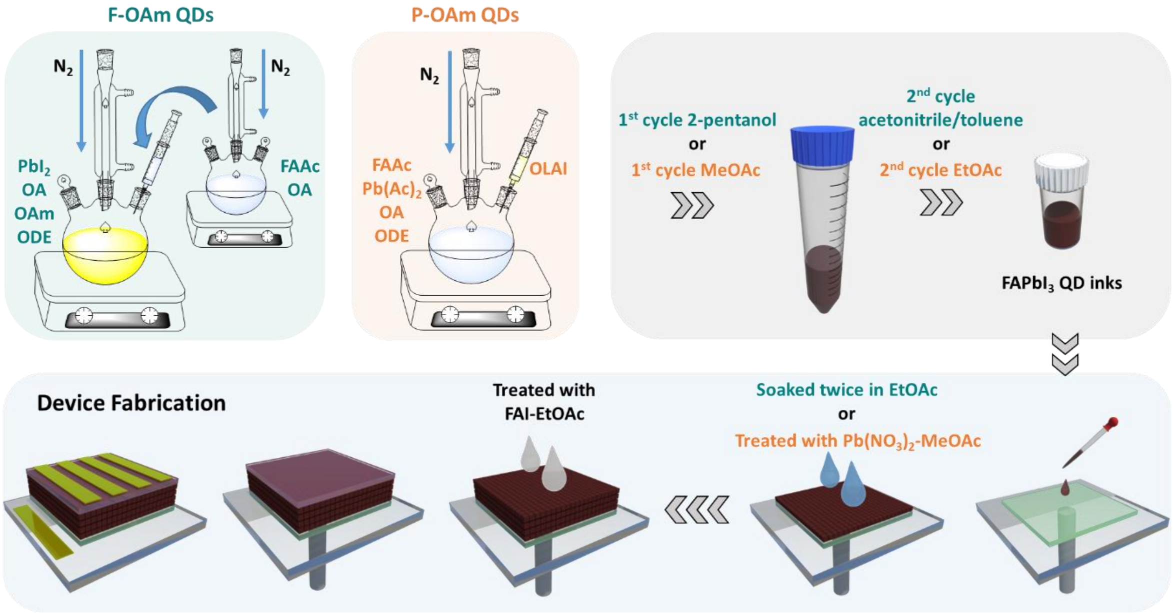

Figure S1. Schematic illustration of the preparation and post-purification processes of $FAPbI_3$ QDs and their corresponding QD devices.

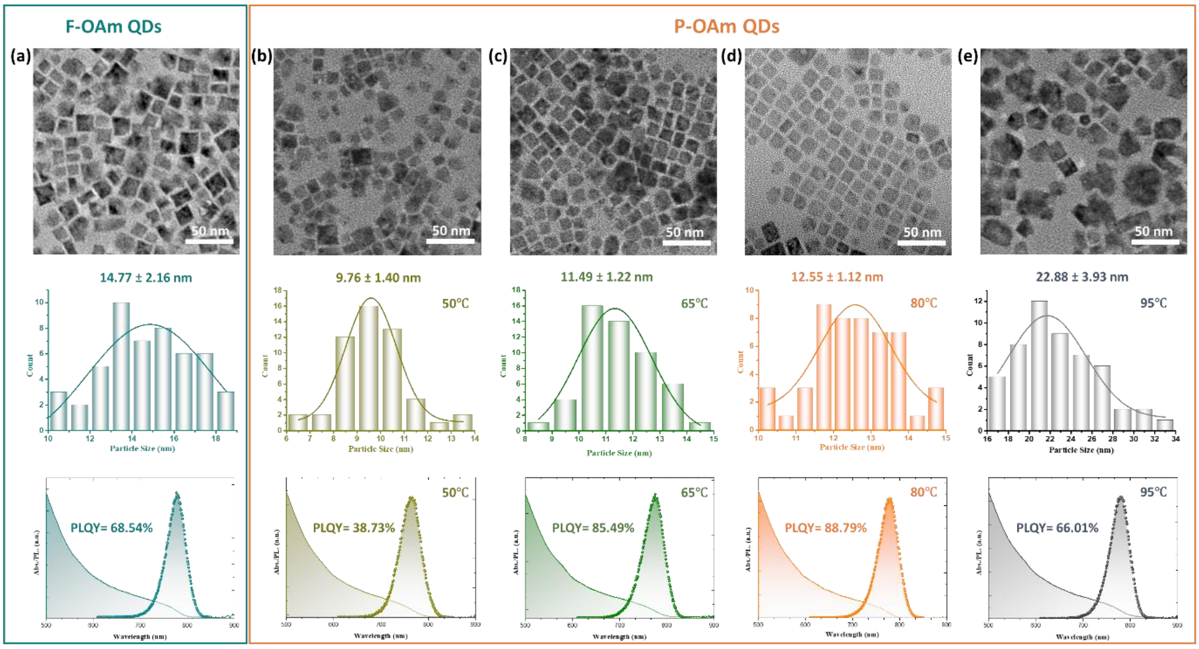

Figure S2. TEM images of (a) F-OAm QDs and P-OAm QDs synthesized at different reaction temperature (b) 50 ℃, (c) 65 ℃, (d) 80 ℃, (e) 95 ℃ and their statistical size distribution diagrams, absorption and PL spectra.

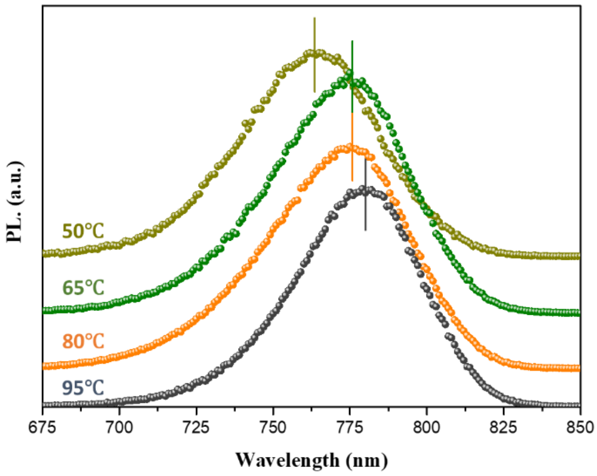

Figure S3. PL spectra of P-OAm QDs synthesized at different reaction temperature.

To investigate the optimum reaction temperature of P-OAm QDs and get more insight into their physicochemical properties, a series of QDs under different reaction temperatures ranging from 50 °C to 95 °C were prepared. As shown in the TEM images in Figure S2, some small particle dots accompanied with a handful of cubic shape QDs can be observed at a low temperature of 50 °C, indicating that low reaction temperature is insufficient to complete the nucleation and growth of QDs. Under the elevated temperatures of 65 °C and 80 °C, uniform $FAPbI_3$ nanocubes can be obtained with their average size (the edge length) increased from 11.49 ± 1.22 nm to 12.55 ± 1.12 nm. However, further increase of the reaction temperature (over 90 °C) cause the anisotropic growth of P-OAm QDs and undesired particle aggregation due to the thermal quenching.[9, 10] As for the optical properties of these P-OAm QDs, we can observe that there is a red-shift of PL peaks with the increase of reaction temperature (Figure S3), illustrating the quantum confinement effect of these P-OAm QDs.[11] Moreover, PLQYs, as a critical evaluation criterion for optical property of QDs, of all samples are also analysed and shown in Figure 1 and Figure S2. It is found that there is a gradually increased PLQYs as reaction temperature goes up, demonstrating that P-OAm QDs with good quality and optical properties prefer to be formed at a relatively higher temperature (above 60 °C) and 80 °C is the optimum reaction temperature for P-OAm QDs.

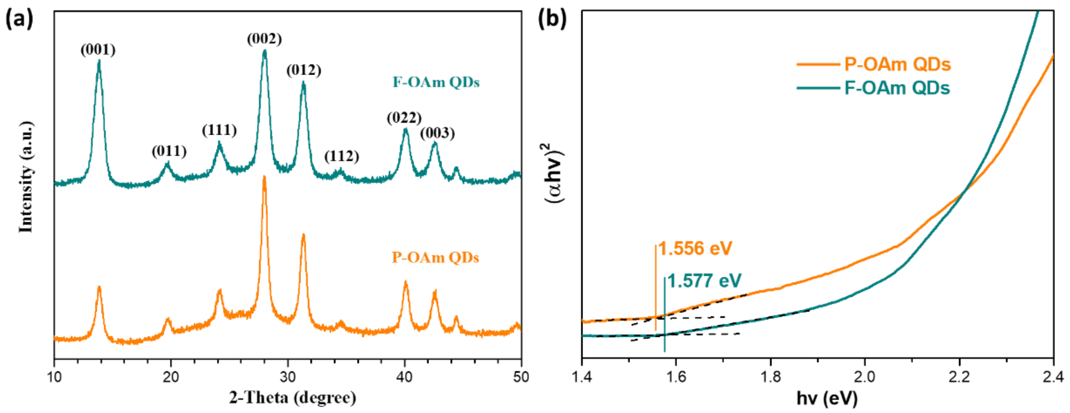

Figure S4. (a) XRD patterns and (b) Tauc plot of absorbance with photon energy (hv) of as-synthesized $FAPbI_3$ QDs.

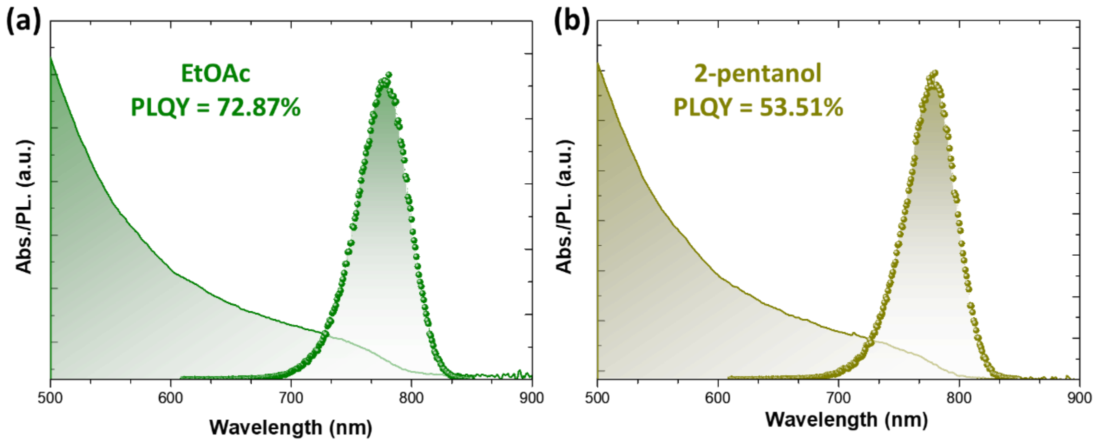

Figure S5. The UV- absorption and PL spectra of P-OAm QDs purified with (a) EtOAc and (b) 2-pentanol as anti-solvents in the first cycle.

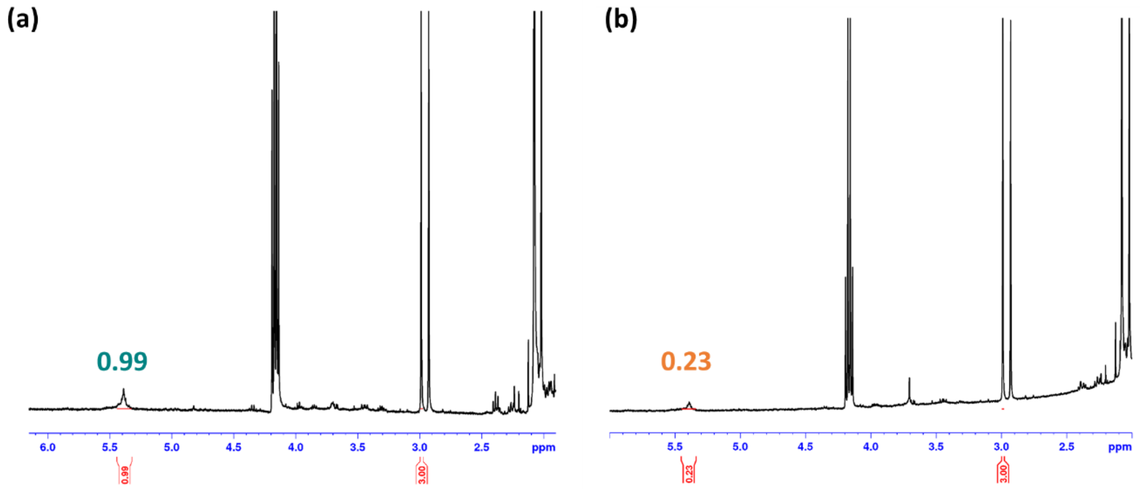

Figure S6. Raw data of the ^{1}H NMR spectra of (a) F-OAm QDs and (b) P-OAm QDs.

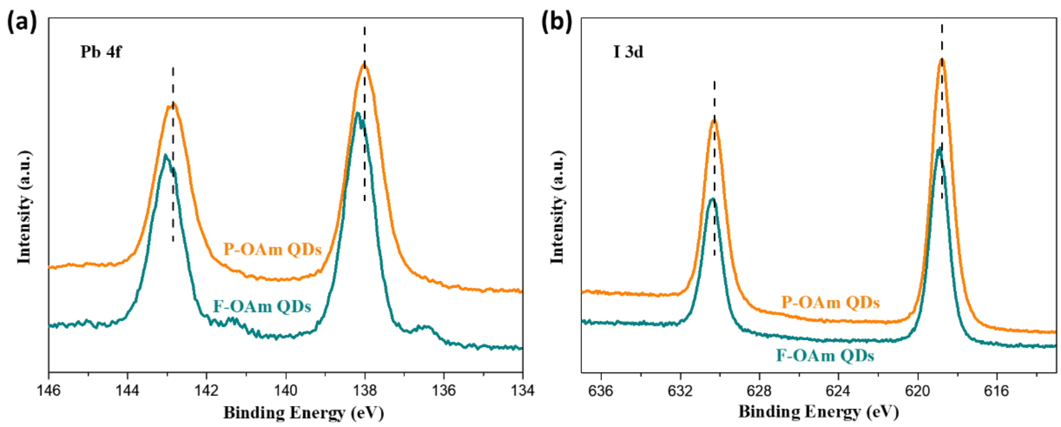

Figure S7. The XPS (a) Pb 4f and (b) I 3d core level spectra of $FAPbI_3$ QD films.

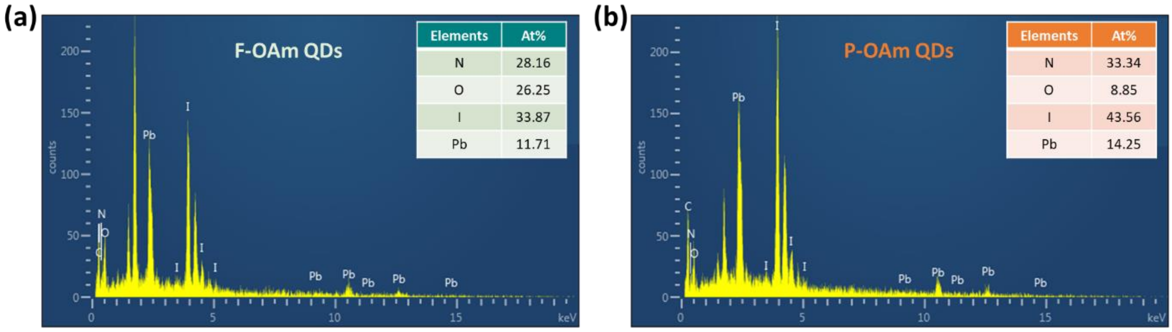

Figure. S8. The EDS patterns of (a) F-OAm QDs and (b) P-OAm QDs.

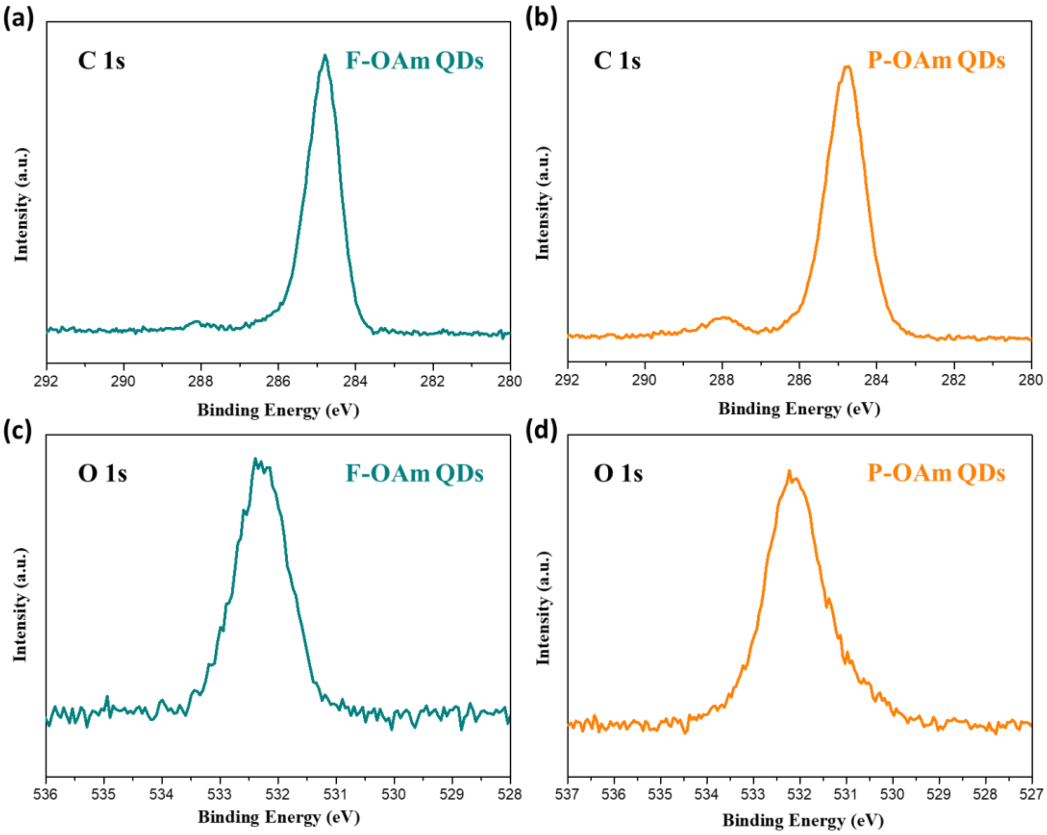

Figure S9. The XPS (a) (b) C 1s and (c) (d) O 1s core level spectra of $FAPbI_3$ QD films.

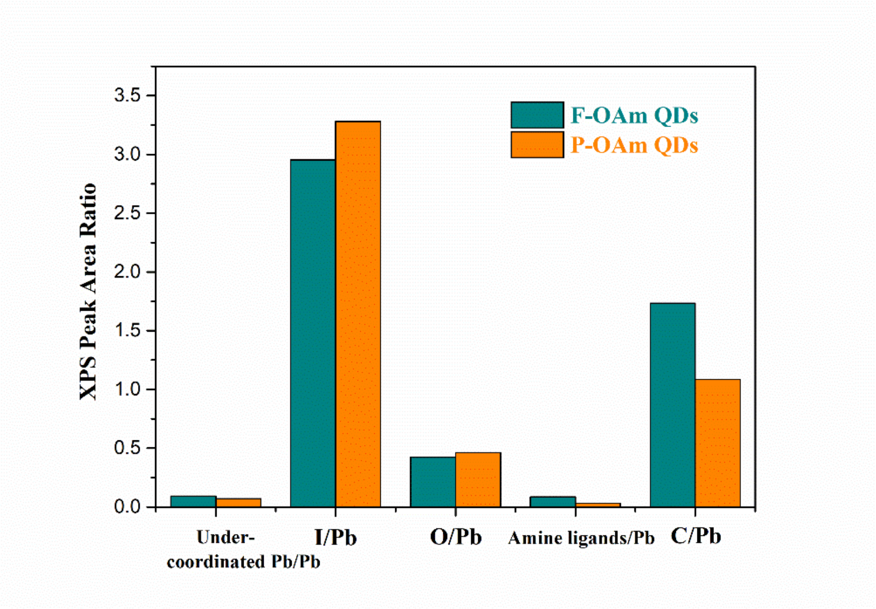

Figure S10. Peak area ratios from the XPS characterization of the $FAPbI_3$ QD films.

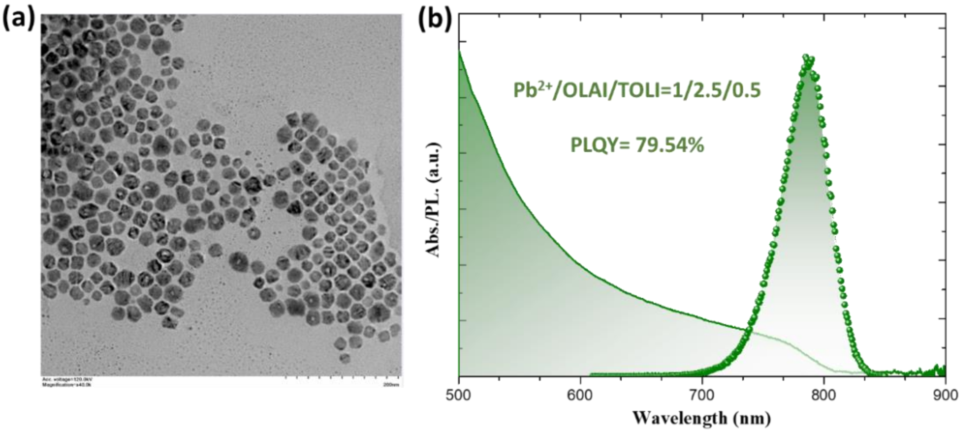

Figure S11. (a) TEM image and (b) UV-absorption and PL spectra of P-OAm QDs prepared with using OLAI and TOLI as halide sources.

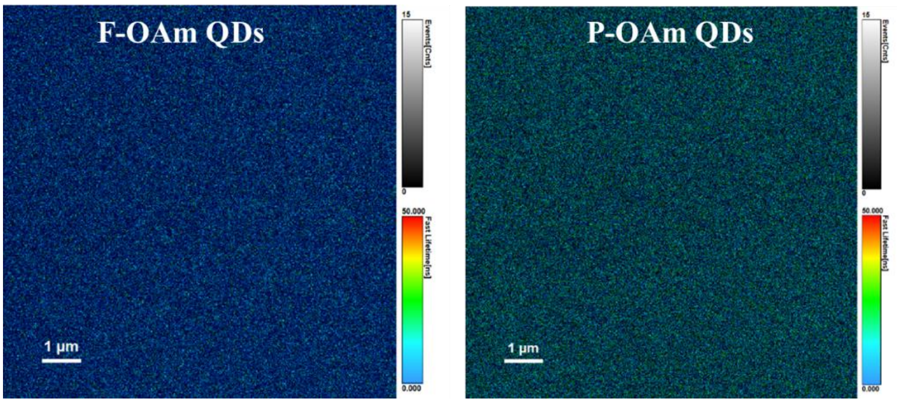

Figure S12. The fluorescence lifetime imaging microscopy images of as-prepared $FAPbI_3$ QD films.

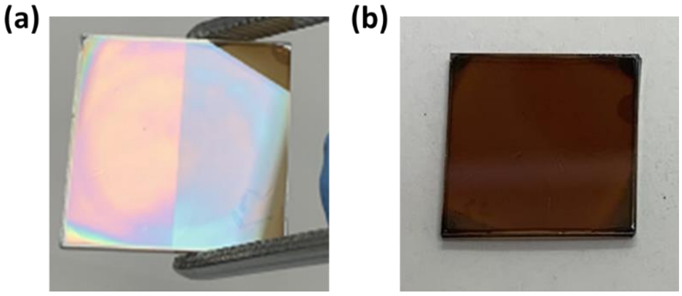

Figure S13. The digital photoes of (a) F-OAm QD and (b) P-OAm QD films treated with $Pb(NO_3)_2$-MeOAc.

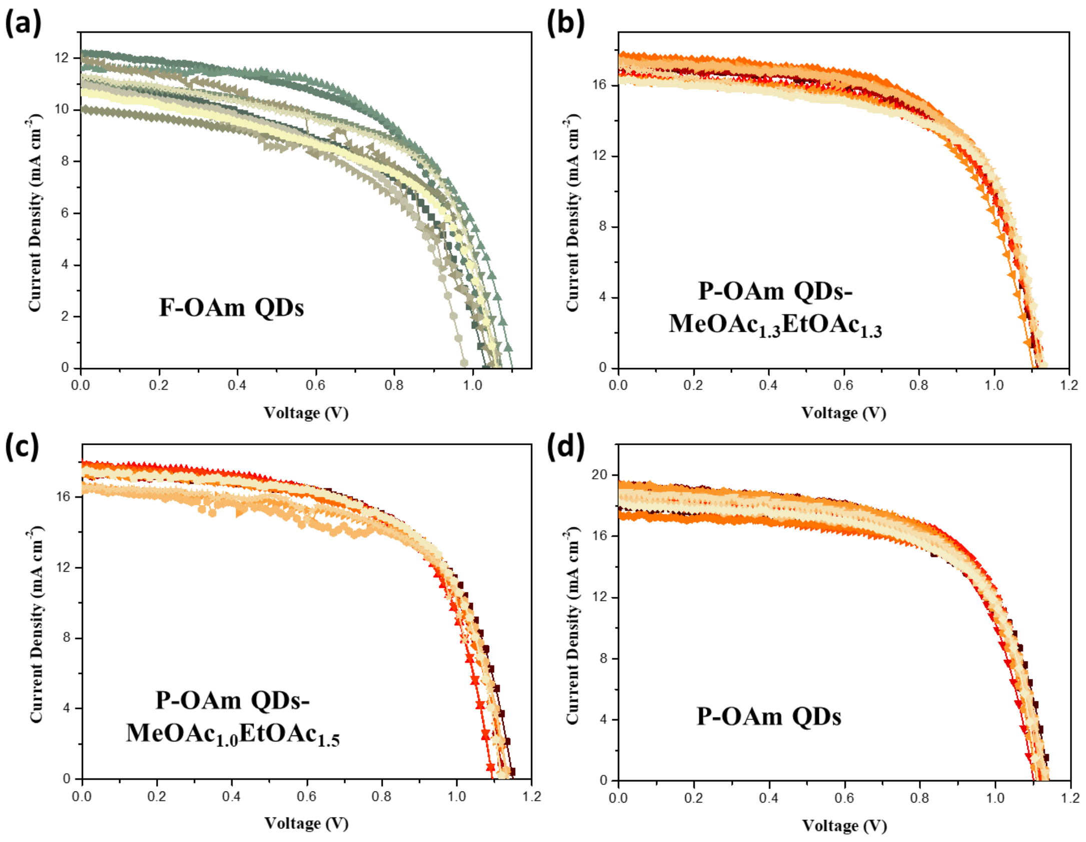

Figure S14. Typical *J*-*V* curves of QDSCs based on as-prepared $FAPbI_3$ QDs: (a) F-OAm QDs (ten devices), (b) P-OAm QDs-$MeOAc_{1.3}EtOAc_{1.3}$ (ten devices), (c) P-OAm QDs-$MeOAc_{1.0}EtOAc_{1.5}$ (ten devices) and (d) P-OAm QDs (sixteen devices).

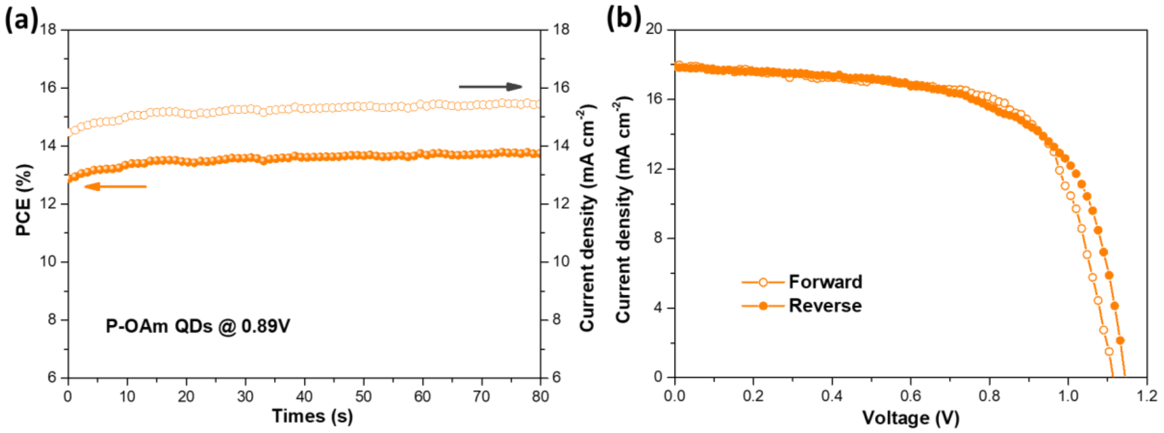

Figure S15. The (a) stabilized power output (measured at the maximum power output of 0.89 V) and (b) *J-V* hysteresis of the P-OAm QD-based device.

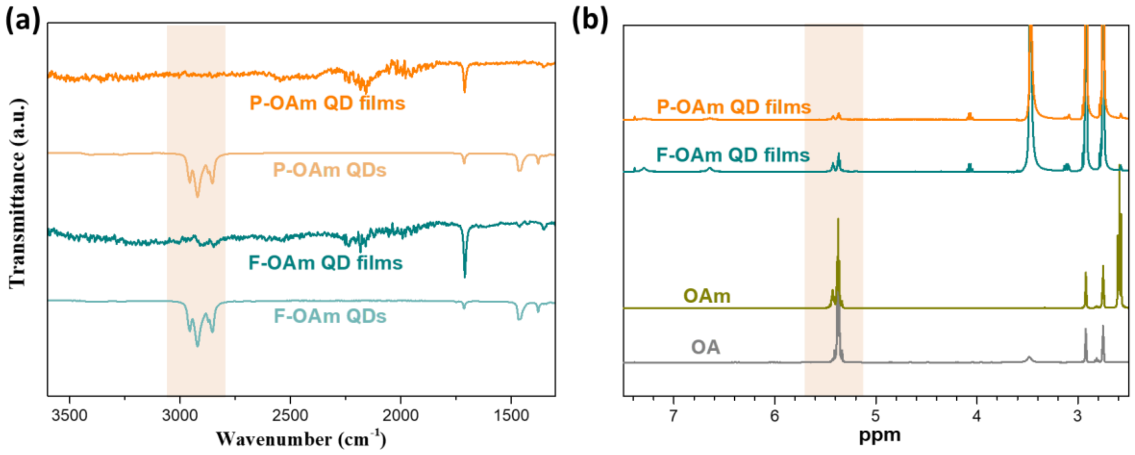

Figure S16. (a) FTIR and (b) ^{1}H NMR spectra of $FAPbI_3$ QDs. The corresponding signals from oleyl species are highlighted with light orange zone, while NMR signals detected at around 4 ppm are attribted to EtOAc served as a quantitation standard.

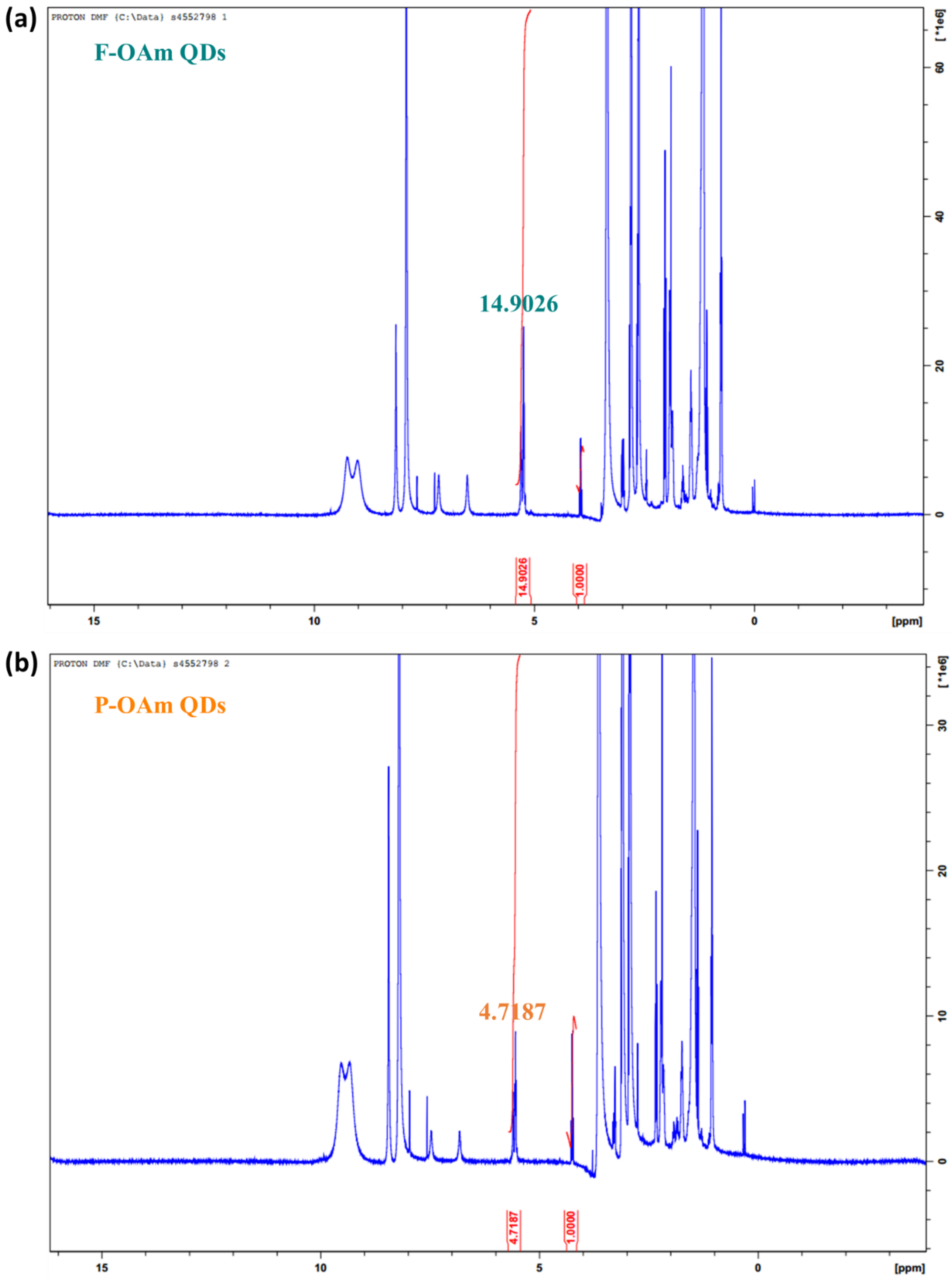

Figure S17. Raw data of the ^{1}H NMR spectra of (a) F-OAm QDs and (b) P-OAm QDs after films deposition.

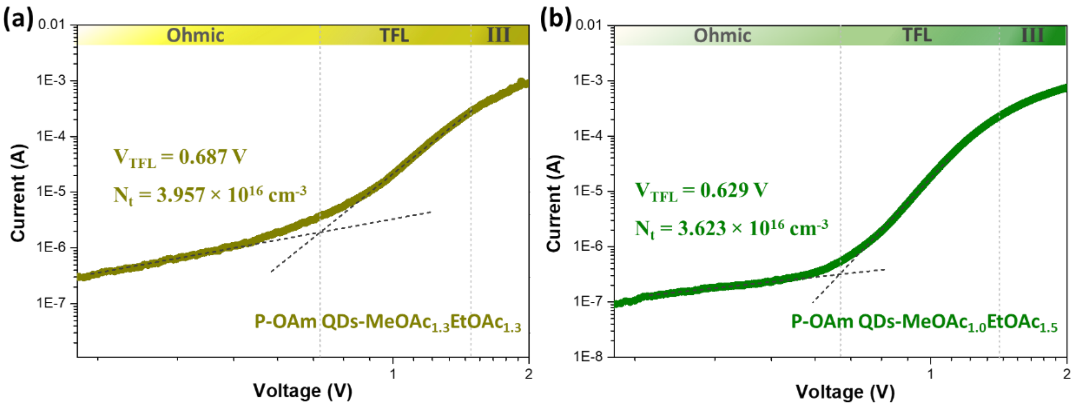

Figure S18. *I-V* curves of electron-only devices (ITO/SnO_2/QDs/PCBM/Au) based on P-OAm QDs purified with different ratio of QDs/anti-solvents.

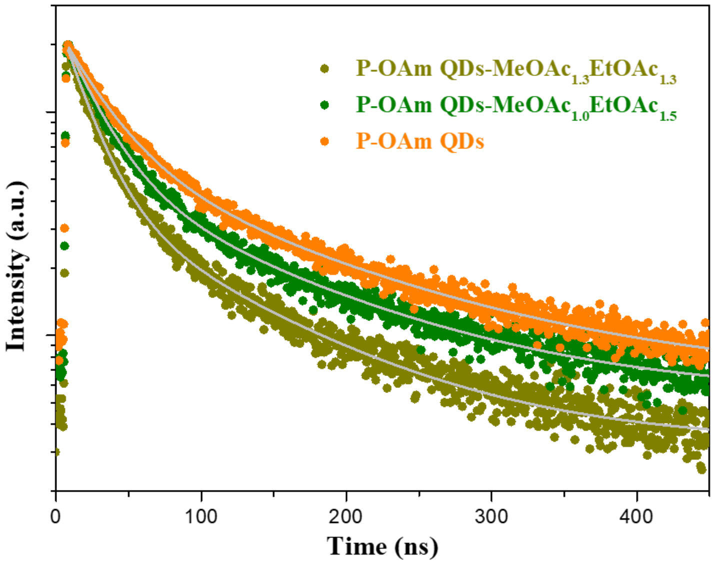

Figure S19. TRPL spectra of P-OAm QD films and their fitted curves.

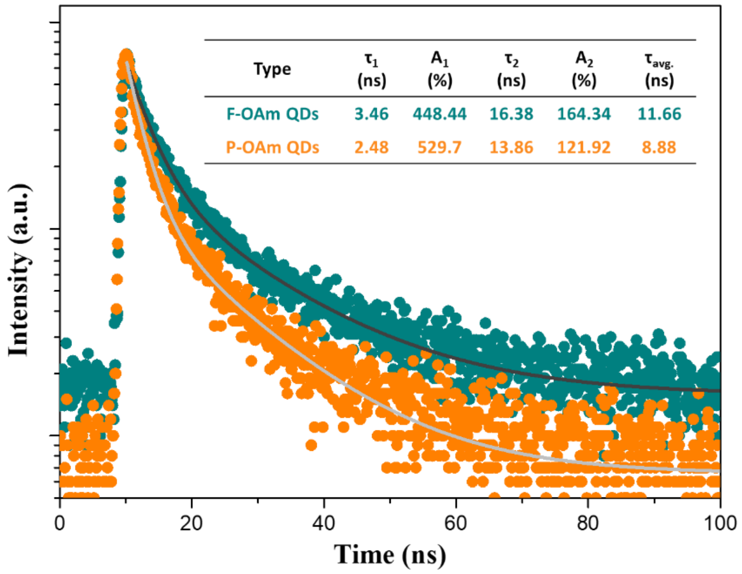

Figure S20. TRPL spectra of $FAPbI_3$ QD films deposited on ITO/SnO_2 substrate.

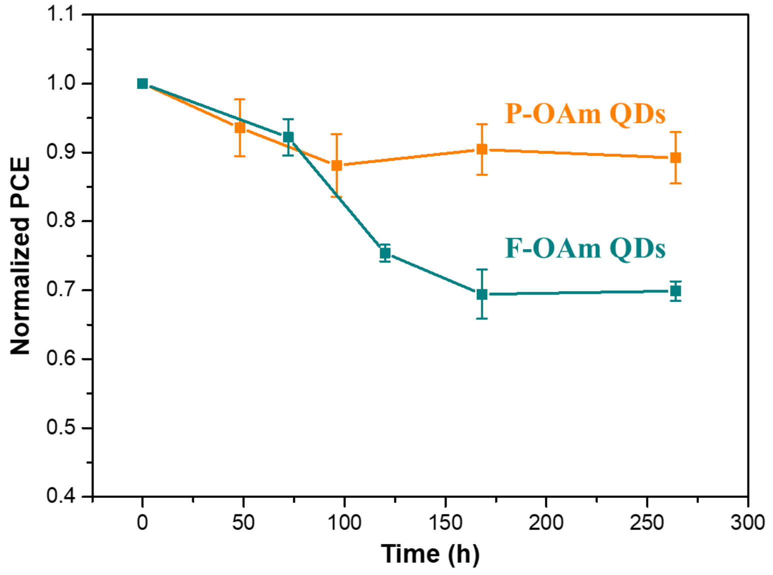

Figure S21. The stability testing of the unencapsulated devices exposing under continuous one-sun illumination in an N_2 atmosphere (LED grow light（Spectrum King MLH140), the device temperature was measure to be 45-60 ℃).

Table S1. Fitting parameters for TRPL decay from as-prepared $FAPbI_3$ QDs.

Type	τ_1 (ns)	A_1 (%)	τ_2 (ns)	A_2 (%)	$\tau_{avg.}$ (ns)
F-OAm QDs	21.73	1474.87	77.45	418.92	49.76
P-OAm QDs - $MeOAc_{1.3}EtOAc_{1.3}$	18.93	1524.49	101.57	375.17	65.96
P-OAm QDs - $MeOAc_{1.0}EtOAc_{1.5}$	24.07	1410.31	121.57	465.19	84.99
P-OAm QDs	29.37	1335.7	138.53	569.68	102.28

Table S2. The formation energy of iodide vacancy in the structure of FA-I and Pb-I termination faces for α-$FAPbI_3$ materials at different potential vacancy sites.

Defect sites	Formation energy (eV)	
	FA-I termination	Pb-I termination
Site 1	2.09	2.07
Site 2	1.96	2.13
Site 3	1.53	2.38

Reference

[1] J. Xue, J.-W. Lee, Z. Dai, R. Wang, S. Nuryyeva, M. E. Liao, S.-Y. Chang, L. Meng, D. Meng, P. Sun, O. Lin, M. S. Goorsky, Y. Yang, *Joule* **2018**, *2* (9), 1866.

[2] H. C. Junqueira, D. Severino, L. G. Dias, M. S. Gugliotti, M. S. Baptista, *Phys. Chem. Chem. Phys.* **2002**, *4* (11), 2320.

[3] M. Grabolle, M. Spieles, V. Lesnyak, N. Gaponik, A. Eychmüller, U. Resch-Genger, *Anal. Chem.* **2009**, *81* (15), 6285.

[4] W. Li, L. Li, H. Xiao, R. Qi, Y. Huang, Z. Xie, X. Jing, H. Zhang, *RSC Adv.* **2013**, *3* (32), 13417.

[5] G. Kresse, J. Furthmüller, *Phys. Rev. B* **1996**, *54* (16), 11169.

[6] J. P. Perdew, K. Burke, M. Ernzerhof, *Phys. Rev. Lett.* **1996**, *77* (18), 3865.

[7] S. Grimme, J. Antony, S. Ehrlich, H. Krieg, *J. Chem. Phys.* **2010**, *132* (15), 154104.

[8] S. Grimme, S. Ehrlich, L. Goerigk, *J Comput Chem* **2011**, *32* (7), 1456.

[9] B. T. Diroll, G. Nedelcu, M. V. Kovalenko, R. D. Schaller, *Adv. Funct. Mater.* **2017**, *27* (21), 1606750.

[10] Y. Zhao, C. Riemersma, F. Pietra, R. Koole, C. de Mello Donegá, A. Meijerink, *ACS Nano* **2012**, *6* (10), 9058.

[11] L. Protesescu, S. Yakunin, M. I. Bodnarchuk, F. Krieg, R. Caputo, C. H. Hendon, R. X. Yang, A. Walsh, M. V. Kovalenko, *Nano Lett.* **2015**, *15* (6), 3692.
